# Electrical‐Stimulation Hydrogel Electronic Skin for Sustainable Hybrid Biomechanical–Electromagnetic Energy Harvesting and Accelerating Wound Healing

**DOI:** 10.1002/advs.75940

**Published:** 2026-06-15

**Authors:** Syun‐Hong Chou, Ming‐Han Lu, Wei‐Chen Peng, Wei‐Chun Yang, Pei‐Yi Liu, Yu‐Hsuan Yang, Yi‐Lin Huang, Cheng‐Hung Tsai, Ting‐Yu Yang, Xuan Zhang, Zhi‐Xian Yan, Ying‐Chih Lai, San‐Yuan Chen

**Affiliations:** ^1^ Department of Materials Science and Engineering National Yang Ming Chiao Tung University Hsinchu Taiwan; ^2^ Department of Materials Science and Engineering National Chung Hsing University Taichung Taiwan; ^3^ Innovation and Development Center of Sustainable Agriculture, i‐Center for Advanced Science and Technology National Chung Hsing University Taichung Taiwan; ^4^ Department of Physics National Chung Hsing University Taichung Taiwan; ^5^ Graduate Institute of Biomedical Sciences China Medical University Taichung Taiwan; ^6^ School of Dentistry, College of Dental Medicine Kaohsiung Medical University Kaohsiung Taiwan

**Keywords:** electrical stimulation, hybrid biomechanical‐electromagnetic energy harvesting, self‐powered electronic skin, triboelectric nanogenerator, wound healing

## Abstract

External electrical stimulation (ES) plays a crucial role in promoting wound healing. However, conventional strategies depend on external power sources and exhibit insufficient responsiveness to the physiological microenvironment, which impedes precise and efficient healing. Herein, a hybrid self‐powered ES electronic skin (HSESES) is designed by integrating a gelatin‐based outer matrix with a zwitterionic conductive inner layer. This electronic skin effectively harvests both biomechanical energy (from body motion) and ambient electromagnetic energy (from surrounding appliances) to provide real‐time and sustainable ES for accelerating tissue regeneration and wound healing. Under physiological conditions, the HSESES can effectively capture biomechanical (33.8 V and 3.2 µW) and electromagnetic energy (±13 V and 7.72 µW). By collecting hybrid energy, the ES generated from the HSESES establishes a safe and stable exogenous electric field that synergizes with the endogenous electric field, thereby enhancing cellular migration, proliferation, and tissue regeneration to facilitate wound repair. In vivo rat studies were confirmed that the HSESES markedly enhanced wound closure. Histological analysis revealed well‐organized epithelial regeneration, collagen deposition, and substantial neovascularization. This study highlights the preliminary potential of HSESES for biomedical applications based on sustainable hybrid energy harvesting and broadens the design paradigm for self‐powered wearable devices.

## Graphical abstract

A hybrid biomechanical–electromagnetic energy‐harvesting e‐skin is developed using a gelatin‐based outer matrix with a zwitterionic conductive inner layer. The e‐skin autonomously generates stable electrical stimulation from daily body motion and ambient electronics, enabling significantly enhanced wound healing bothin vitro and in vivo. The sophisticated design establishes a sustainable and biocompatible strategy for next‐generation self‐powered bioelectronic medical systems.

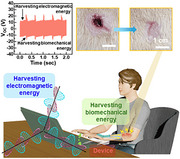

## 1 Introduction

Despite more than a century of research and clinical advances, wound healing remains a formidable challenge for global healthcare and regenerative medicine. Owing to their complex and dynamic physiological mechanisms, minimal disturbances may impede repair and lead to chronic wounds or severe complications such as diabetic foot and venous ulcers [1, 2, 3, 4, 5]. Inflammation, proliferation, and remodeling are the sequential and overlapping physiological phases of tissue repair. However, current therapeutic strategies, such as wound debridement, bio‐adhesives, wound dressings, and drugs, fail to meet the dynamic demands of the multiple healing stages, potentially resulting in surgical complications, drug toxicity, and drug resistance [6, 7, 8]. Electrical stimulation (ES) is a non‐invasive and non‐pharmacological therapeutic strategy capable of regulating physiological repair processes [9, 10]. By modulating Na^+^/K^+^ ion transmembrane migration and promoting growth factor expression, ES induces cell proliferation, migration, and tissue regeneration, effectively mimicking and enhancing the healing response driven by the endogenous electric field. Moreover, ES exhibits superior biocompatibility and minimal immunogenicity during therapy [11, 12]. However, current ES approaches are limited by device bulkiness, reliance on external power supplies or batteries, and the need for expert operation. Therefore, miniaturized, self‐powered, and flexible ES‐generating apparatuses with sustainable and biocompatible performance are required for next‐generation intelligent wound care technologies [13, 14, 15, 16, 17].

Triboelectric nanogenerators (TENGs) operate through the synergistic coupling of contact electrification and electrostatic induction, effectively converting diverse mechanical energies into electrical energy [18, 19]. Owing to their high energy conversion efficiency, wearability, flexibility, and low cost, TENGs provide a promising self‐powered solution for ES devices, ensuring safety, comfort, and continuous operation, while eliminating reliance on external energy sources [15, 16]. Recently, TENGs have been integrated into ES therapy, providing a promising self‐powered strategy to facilitate wound repair [15, 16, 17, 20]. Jeong et al. presented a wearable, stretchable ionic TENG as a patch to accelerate wound healing [21]. Venkatesan et al. reported a TiO_2_–MXene‐incorporated polystyrene nanofiber‐based TENG for enhancing wound healing [22]. In addition, integrating other stimuli such as ultrasound or photothermal therapy with ES treatment is promising for improving wound‐healing efficacy [23, 24, 25]. Despite encouraging advancements, these strategies still face several limitations, including infection risk, mechanical flexibility, and restricted in‐situ applicability. In particular, the inherent dependence on continuous and intense body motion to drive power generation may cause secondary damage and compromise the stability. Thus, integrating TENG with an additional energy‐harvesting approach is required to overcome the intrinsic constraints of TENG for meeting the clinical needs of ES therapy.

Harvesting ambient electromagnetic (EM) energy, which is an omnipresent and sustainable energy source in modern environments, is a promising strategy. The efficient harvesting and conversion of ambient EM waste energy enhances the accessibility and stability of self‐powered systems and alleviates the environmental impacts of EM pollution [26, 27]. Although the technological development of EM energy collection has been extensively investigated, applications of EM energy harvesting in biomedical wound repair remain scarce [28, 29]. Moreover, developing materials that can efficiently harvest biomechanical (BM) and EM energy while maintaining biocompatibility to meet the demands of human clinical use remains challenging.

Herein, we present a hybrid self‐powered ES electronic skin (HSESES) that simultaneously harvests BM energy and surrounding EM energy for sustained and efficient ES. This flexible electronic skin (e‐skin) is engineered with a sandwich‐structured design comprising gelatin‐based outer layers and a zwitterionic polymer core. To the best of our knowledge, this study is the first to integrate BM and EM energy harvesting using the HSESES, offering a sustainable strategy for efficient wound repair. The HSESES achieves outputs of 33.8 V and ±13 V from BM and EM energy harvesting, respectively, addressing key limitations of conventional TENGs and broadening their biomedical applicability. Additionally, preliminary evaluations suggest the potential biocompatibility of the device. Notably, comprehensive in vitro and in vivo results demonstrated that hybrid‐source ES provided superior therapeutic outcomes in wound repair. Collectively, the HSESES exhibited reliable energy‐harvesting capability and remarkable therapeutic efficacy in biomedicine. The superior wound‐healing responses elicited by the hybrid‐source stimulation validated the practical potential of this e‐skin for next‐generation sustainable self‐powered bioelectronic medical devices.

## 2 Results and Discussion

### 2.1 Design, Fabrication, and Characterization of the HSESES

**FIGURE 1:**
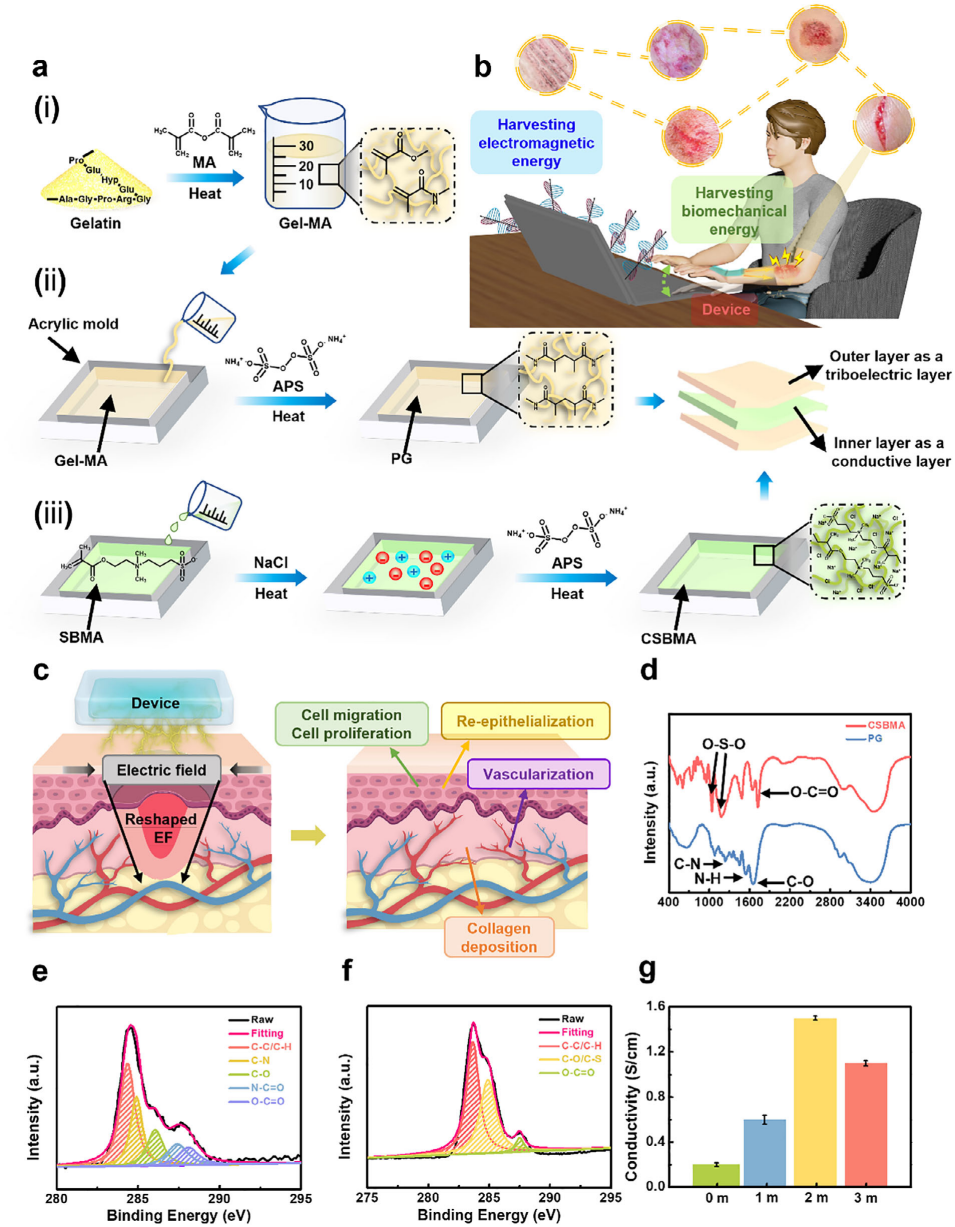
Overview concept and material design of the HSESES and its potential wound‐healing application. (a) Schematic of fabrication steps of the HSESES and (b) demonstration of BM and EM energy harvesting to generate ES applied in wound healing. (c) Mechanism of ES in promoting wound healing. (d) FTIR spectra of various materials. C 1s spectra of (e) PG and (f) CSBMA. (g) Conductivity of the conductive CSBMA.

Figure 1a illustrates the fabrication process of the HSESES. First, gelatin was functionalized with MA to introduce C═C bonds and subsequently crosslinked using APS‐initiated radical polymerization to construct a bioactive outer layer. SBMA and salts were then uniformly mixed and further polymerized under APS induction to form a conductive polymeric inner layer. Finally, integrating the layers yielded a flexible and stable sandwich‐structured device. The detailed manufacturing process is presented in the Experimental Section. Figure 1b presents the application scenarios of the HSESES. Through the synergistic harvesting of BM (from human motion) and EM (from adjacent appliances) energy, the e‐skin achieves sustained self‐powered ES for accelerating wound healing. The corresponding therapeutic mechanism of ES derived from the HSESES involves the coordinated modulation of the wound microenvironment (Figure 1c). The generated electrical energy regulates multiple biological pathways and optimizes the wound microenvironment to accelerate healing [11, 12, 16, 17].

To verify the successful synthesis of each material in the HSESES, the chemical structure was systematically investigated through Fourier transform infrared (FTIR) and X‐ray photo‐electron spectroscopy (XPS) analyses, as shown in Figure 1d–f. In the FTIR spectrum, the characteristic peak of CSBMA at 1722 cm^−1^ was related to the ester group (C = O stretching), and the absorption peaks at 1176 and 1042 cm^−1^ originated from the sulfonate group (O‐S‐O stretching) [30, 31, 32]. The peaks of PG at 1238, 1538, and 1636 cm^−1^ were attributed to amide‐related C‐N, N‐H, and C═O bonds, respectively [33, 34]. The XPS analysis confirmed these findings. In the C 1s spectrum, the peaks at 287.4 and 288.1 eV were attributed to the formation of the N‐C═O and O─C═O bonds in PG, respectively, whereas the signal at 284.9 eV was attributed to the C‐O/C‐S bonds in the CSBMA [35, 36, 37, 38]. NMR analysis further confirmed the successful synthesis of the polymeric network (Figure S1). Building on the above validations, the electrical behavior of the zwitterionic polymer core was investigated to clarify its dependence on the ionic composition and conductivity (Figure 1g). As expected, the conductivity significantly increased up to 1.5 S cm^−1^ at 2 m, revealing the efficient ion transport within the polymer. However, a slight decrease in conductivity was observed at 3 m, which could be attributed to the aggregation of oppositely charged ions, thereby decreasing the number of free charge carriers and reducing the overall conductivity [39]. To satisfy e‐skin applications, the mechanical properties of the HSESES were comprehensively investigated. The device demonstrated robust strength and excellent deformability under various relative humidity (RH) conditions (Figure S2a) [40, 41, 42, 43]. Adequate adhesion and stable performance over repeated cycles were confirmed by porcine skin‐based assays (Figure S2b–d) [44, 45, 46]. Notably, biocompatibility studies indicated negligible cytotoxicity (Figure S3).

### 2.2 Performance of the HSESES

**FIGURE 2:**
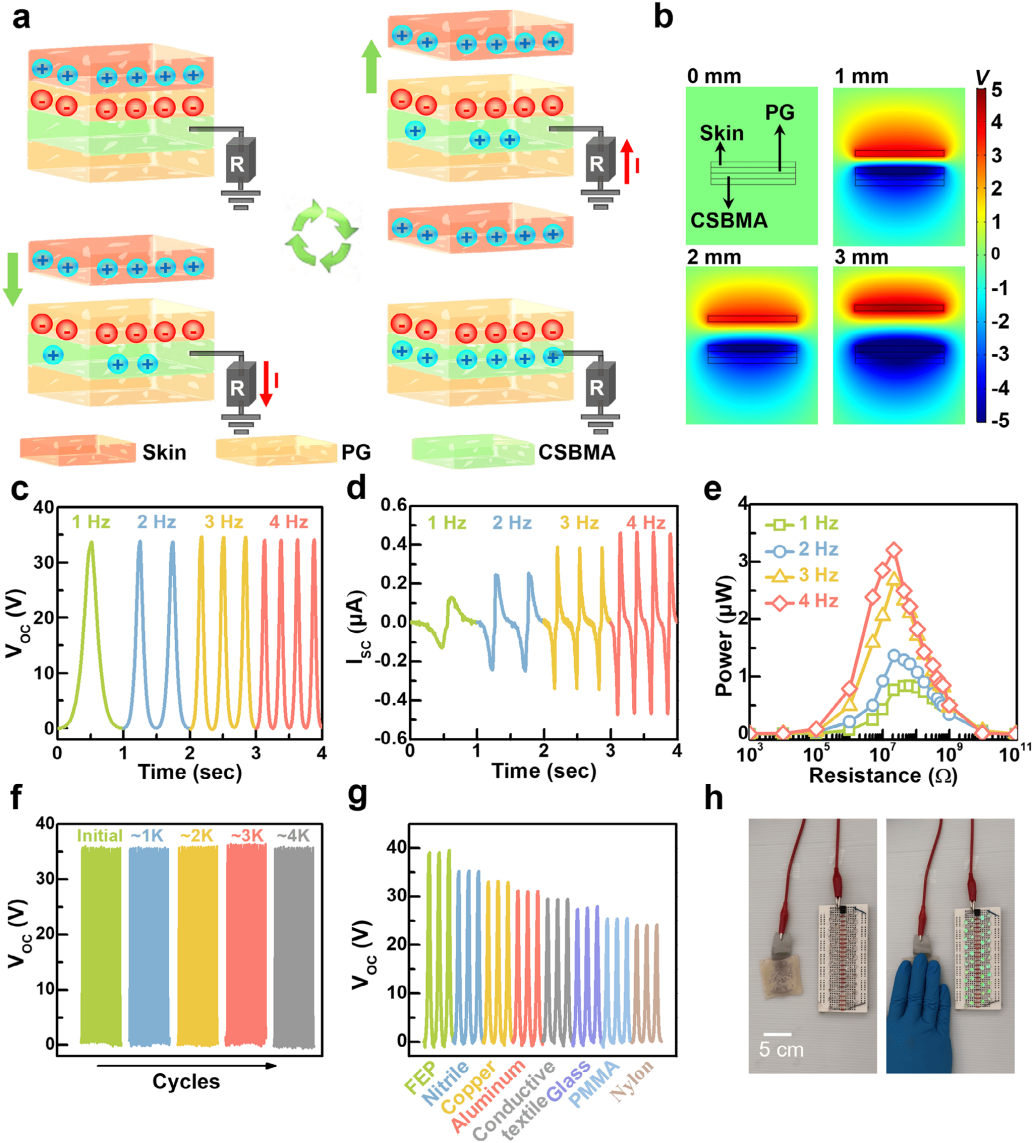
Electrical output performances of the HSESES by harvesting mechanical energy. (a) Working principle of the HSESES. (b) Stimulated electric potential distribution with open‐circuit condition during contact and separation process between skin and the HSESES. Outputs of (c) *V*_oc_ and (d) *I*_sc_ of the HSESES. Relationships of outputs of (e) power of the HSESES on different external load resistances. (f) *V*_oc_ during a cyclic operation. (g) *V*_oc_ for various contact materials. (h) Photos of powering LEDs by the HSESES.

Figure 2a illustrates the operation mechanism of the HSESES in single‐electrode mode [47, 48]. Electrification occurs at both surfaces as the skin contacts the HSESES and generates the same amount but opposite polarity charges. The PG layer was negatively charged, whereas the skin was positively charged, with no electrical potential difference. Upon separation, a potential difference is generated, which induces the flow of electrons from the HSESES electrode to the ground to maintain a new electric neutrality. When the skin approaches the HSESES, a negative potential difference is generated, causing electron flow in the opposite direction to balance the electric neutrality. Periodically repeating these processes creates continuously alternating electricity sources. Figure 2b depicts the COMSOL simulations of the electric field during the contact and separation processes under open‐circuit conditions. The relative motion of the two surfaces induces a potential difference and drives the current flow.

A comprehensive analysis was conducted for the HSESES (5 × 5 cm^2^; 3 mm thickness) by applying a 3 N contact force (Figure 2c–g and Figures S4–S8). As the contact frequency increased from 1 to 4 Hz, open‐circuit voltage (*V*_oc_) and transferred charges (Q_tr_) remained relatively constant at 33.8 V and ‐13 nC, respectively (Figure 2c and Figures S4 and S5). The short‐circuit current (I_sc_) increased from 0.13 to 0.46 µA due to a fast charge transport caused by a high frequency in Figure 2d. Subsequently, the output performances of the HSESES as a power source to drive various external resistances were evaluated (Figure 2e and Figure S6). To define the optimal power at various frequencies (Figure 2e), the output power values for various resistances were calculated using the formula P = IV, where P, I, and V represent the power, current, and voltage, respectively [49, 50]. A maximum power value of 3.2 µW was obtained at a resistance of 22 MΩ and a frequency of 4 Hz. Cyclic testing revealed no noticeable degradation in V_oc_, Q_tr_, or I_sc_ after 4000 cycles, indicating excellent long‐term stability. (Figure 2f and Figure S7). Figure 2g and Figure S8 show the effects of various contact materials on the V_oc_ and I_sc_ of the HSESES, respectively, demonstrating its broad applicability. In addition, the electricity generated from mechanical energy harvesting was used to directly power 42 light‐emitting diodes (Figure 2h). To mimic practical e‐skin application scenarios, the outputs were evaluated under different RH conditions and further discussed in Figure S9. Moreover, BM energy harvesting at different body locations was investigated (Figure S10).

Beyond directly driving external loads, the HSESES can be integrated with a rectifier and capacitor to create an equivalent circuit, enabling flexible charge‐discharge management and broadening practical applicability, such as the integration of wearable devices (Figure S11a). Detailed analyses are provided in Figure S11. In summary, these demonstrations reveal the energy‐harvesting potential of the HSESES in wearable miniaturized devices.

**FIGURE 3:**
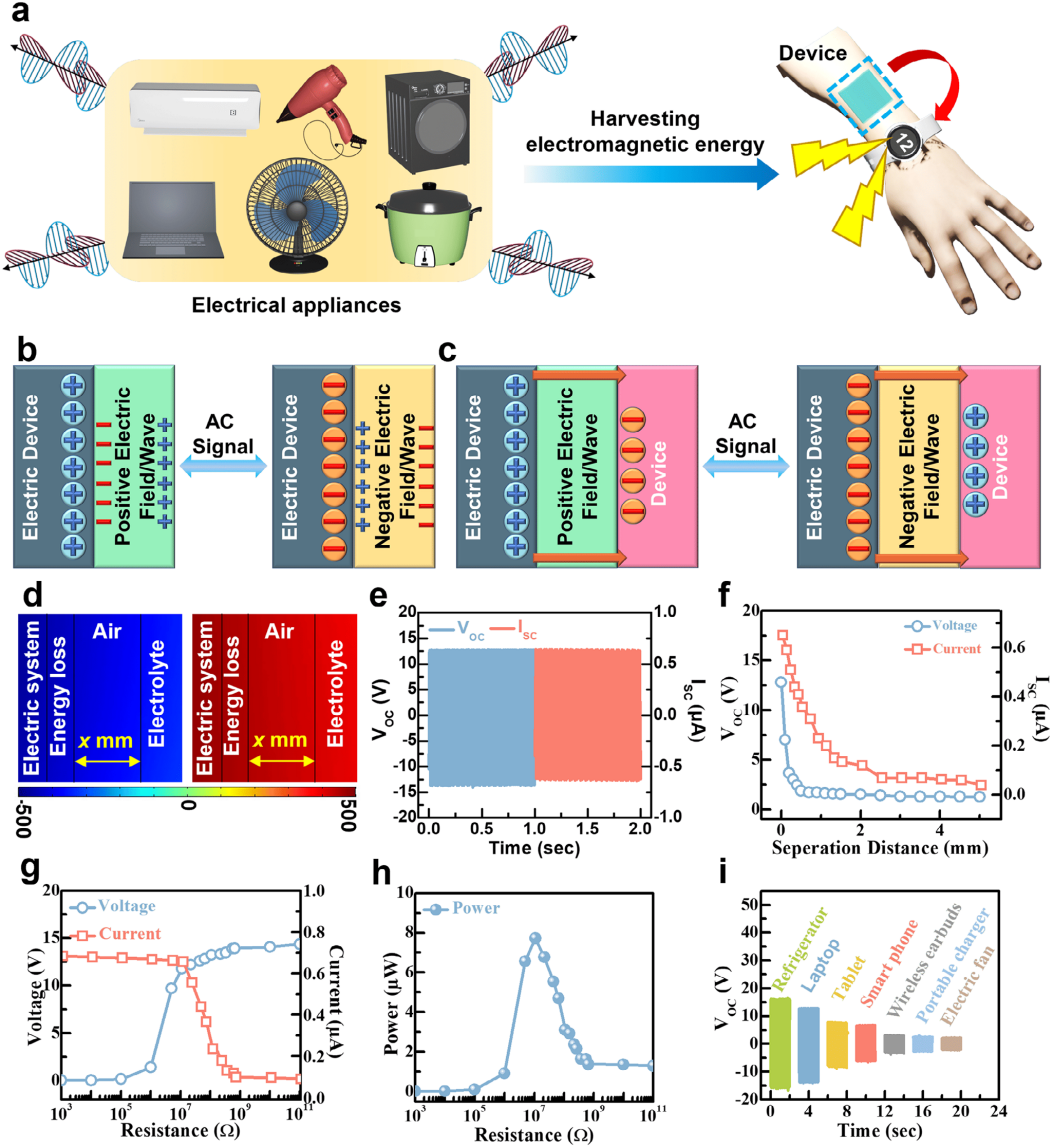
Using the HSESES to harvest EM energy from surrounding appliances. (a) Schematic of concept and corresponding application of converting EM energy released from surrounding electronics into useable energy. Schematic of the mechanisms of (b) dielectric loss and (c) scavenging EM energy using the HSESES. (d) Stimulation of electric potentials by the HSESES when approaching electronics within a specific distance. Generated outputs of (e) *V*_oc_ (blue) and I_sc_ (red) of the HSESES laid on a laptop. (f) Dependence of outputs on separation distances between the HSESES and a laptop. Dependence of (g) voltage and current and (h) power of the HSESES on the external load resistances. (i) *V*_oc_ of scavenging EM energy dissipated from various electrical appliances by the HSESES.

Considering the unstable factors of harvesting mechanical energy using TENGs, such as skin humidity, activity frequency, and contact pressure, incorporating another harvesting method that works synergistically with TENGs can improve the practical efficiency. The HSESES converts environmental EM waves emitted by nearby electrical appliances into usable electricity. Figure 3a depicts the concept and the application scenarios. EM waves originate from various AC electronics that are used daily and inevitably lose energy during operation, in ways such as thermal dissipation. This energy loss arises from the interactions between nearby dielectric materials and alternating electric fields derived from electronics [28, 51]. A previous study employed a stretchable liquid–metal fiber to convert EM energy into usable electricity [28]. The operation mechanism leverages the induction of an alternating electrostatic potential to scavenge the dissipated EM energy to power electronics, as shown in Figure 3b,c. As the HSESES approaches the operating appliance, the alternating electrostatic potential in the electrode of the HSESES is induced by the alternating electric field produced by the appliance. Thus, the free electrons in the electrode periodically redistribute depending on the alternating electrostatic potential variation, subsequently generating electricity for further uses.

The relationship between the electric potential and AC‐powered devices was further explored through finite element simulations, as illustrated in Figure 3d and Figures S12 and S13. For theoretical analysis, positive/negative charges on the electrical devices were presumed to stimulate the generation of induced alternating electric fields. Notably, the induced electrostatic potential increased with decreasing distance between the HSESES and electrical devices. In practice, to harvest EM energy, the HSESES was positioned on a laptop connected to a 60 Hz AC power outlet (Figure 3e). The outputs were quantified by *V*_oc_ and *I*_sc_, reaching ±13 V and ±0.65 µA, respectively. To ensure measurement reliability, grounding and shielding were carefully controlled to exclude electrostatic artifacts (Figure S14). The output performances sharply declined with the increasing distances between the HSESES and a laptop (Figure 3f and Figure S15). The outputs were maintained at ±1.52 V and ±0.12 µA for a 2‐mm separation, verifying the ability of the non‐contact energy harvesting attribute. The effects of various external loads on the voltage, current, and power are shown in Figure 3g,h. A maximum output power of 7.72 µW was observed at 11 MΩ. Interestingly, the energy output generated from EM pollution occurred at a significantly higher working frequency (60 Hz) than that generated by human mechanical motions (≤ 4 Hz). This characteristic mitigates the low‐frequency limitation of TENGs, thereby broadening the collection range and enhancing energy conversion efficiency. To evaluate practical applicability, device durability was examined over a 7‐day operation (Figure S16). Outputs under various RH conditions were investigated to mimic e‐skin environments (Figure S17).

To further align with daily life, the HSESES was used to harvest EM energy from various surrounding electronics, such as refrigerators, tablets, smartphones, wireless earbuds, portable chargers, and electric fans. All electronics were connected to the main power supply. The corresponding output performances are shown in Figure 3i and Figure S18, verifying the feasibility of the HSESES. The EM radiation intensity varies across electronic devices, resulting in different amounts of electricity being converted by the HSESES. The applicability of harvesting EM energy to drive electronic devices is further discussed in Figure S19. For practical application, the device was attached to the wrist to mimic daily activities such as typing while harvesting EM energy from a laptop. (Figure S20). These discoveries highlight its potential as a self‐powered source for biomedical applications, including wound healing.

**FIGURE 4:**
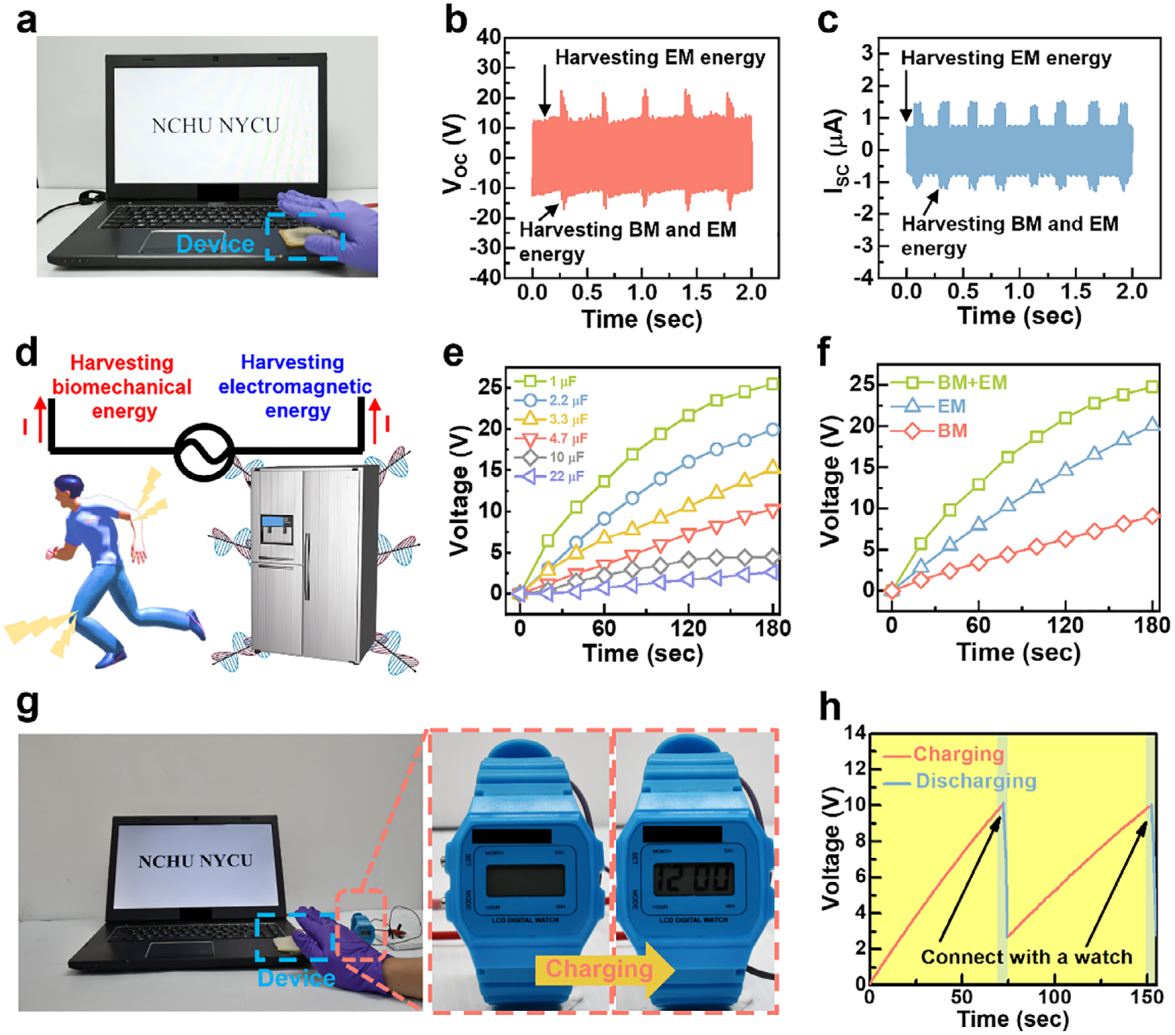
Output performances of the HSESES for harvesting both BM and EM energy. (a) Photos of the HSESES positioned on a laptop for harvesting BM energy by hand contact and EM energy from the operating laptop. Generated outputs of (b) *V*_oc_ and (c) *I*_sc_. (d) Schematic of the HSESES transforming hybrid energy into the electricity. (e) Charging curves for various capacitors. (f) Comparison of charging performances of three power supply strategies for charging a 1 µF capacitor. (g) Photos of harvesting hybrid energy for powering an electronic watch, and (h) its real‐time charging/discharging curves.

To systematically evaluate multi‐source energy harvesting, the HSESES was positioned on a laptop and subjected to the mechanical energy from hand contact (Figure 4a,d). Simultaneous harvesting of biomechanical (BM) and EM energy generated V_oc_ of 35 V and I_sc_ of 2.5 µA (Figure 4b,c). Figure 4e displays charging curves of capacitors with varying capacitances under hybrid energy harvesting. The 1 µF capacitor was charged to 25.4 V within 180 s. The results suggest that both energy sources can be collected simultaneously and independently. Notably, accumulated electrical energy enabled faster charging, with the 1 µF capacitor reaching 13 V (hybrid), 7.9 V (EM), and 3.4 V (BM) within 60 s (Figure 4f). Figure 4g, Figure S21a and Movies S5 and S6 illustrate the practical charging of electronics through hybrid energy harvesting, and the corresponding charging/discharging curves are shown in Figure 4h and Figure S21b.

### 2.3 ES From the HSESES for Accelerating Wound Healing

**FIGURE 5:**
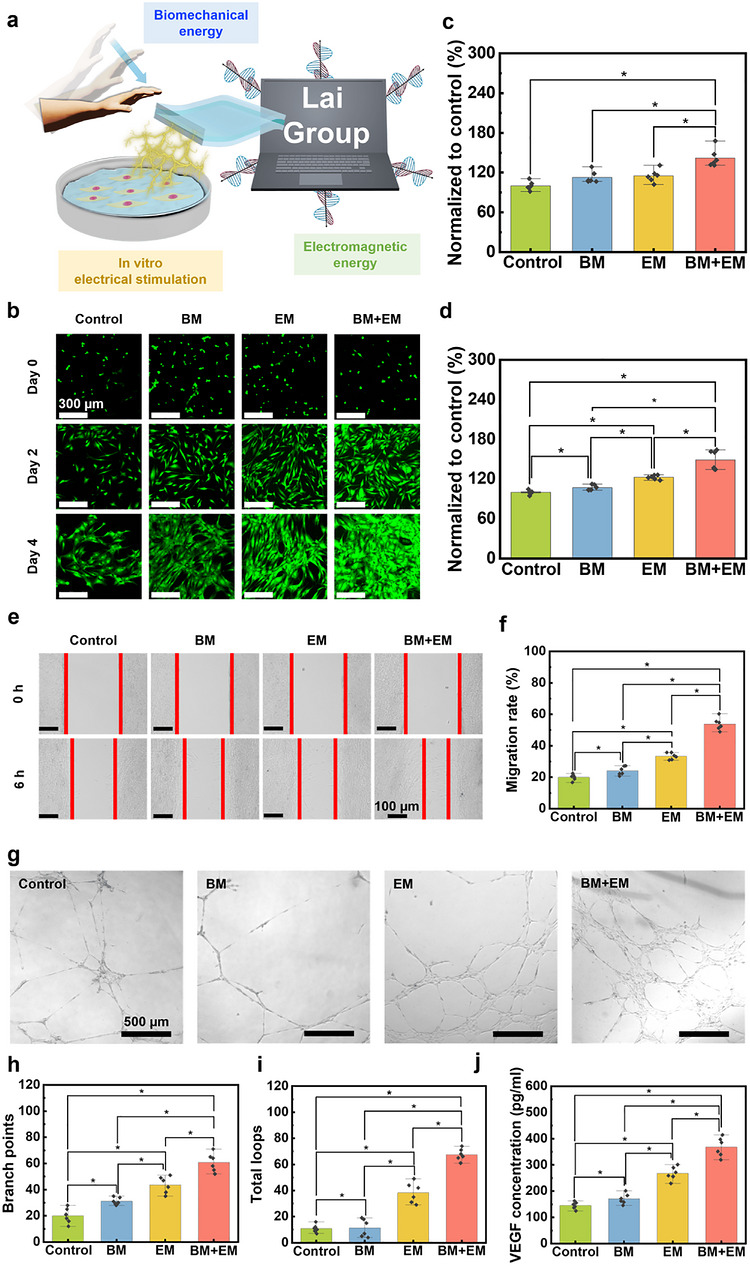
Influence of ES on in vitro cellular behaviors. (a) Schematic of the HSESES for applying ES on cells. (b) Live/dead cell staining images after ES with the HSESES recorded on days 0, 2, and 4. The corresponding cell viability on (c) day 2 and (d) day 4 (*n* = 6, significance indicated by *p < 0.05). (e) Cell migration images after 6 h of ES with the HSESES. (f) The corresponding cell migration ratios of the control and ES groups (n = 6, significance indicated by *p < 0.05). (g) Tube formation assay of ES‐treated HUVECs, visualized after 6 h of incubation. The corresponding results of (h) branch points, (i) total loops, and (j) VEGF concentrations.

Exogenous electric fields potentiate endogenous bioelectric effects, facilitating cell proliferation and migration, thereby accelerating wound healing [11, 52]. With potential biocompatibility, the multi‐source energy‐harvesting HSESES serves as a self‐powered ES source for continuous and controlled therapeutic intervention, offering a promising treatment strategy. To assess the effect of ES from the HSESES on wound healing, in vitro fibroblast cell tests were conducted, as shown in Figure 5a. In a standard experimental setup, the following three energy conversion strategies were utilized as the ES sources: i) BM energy harvesting, ii) EM energy harvesting, and iii) hybrid BM and EM energy harvesting. Fibroblasts’ viability was assessed with and without ES. As shown in Figure 5b, the significant increase in cells under all ES strategies at 48 h confirms the beneficial impact on cell proliferation. This effect is attributed to ES‐induced intercellular communication and mass transport, regulating the cellular proliferation and differentiation behaviors [23]. In particular, cells in the ES groups (BM, EM, and BM+EM) showed the 1.1‐fold, 1.2‐fold, and 1.5‐fold increases in cell proliferation on 4 days, respectively, relative to those in the control group (Figure 5c,d). Interestingly, the cells subjected to ES in all stimulation groups exhibited more contractile behavior than those in the control group. In addition, the electrically stimulated cells grew and aligned along the direction of the electric field, whereas the unstimulated cells displayed random growth. Such directional alignment is consistent with electro‐taxis, where electric fields guide cell migration [53]. A scratch assay was further performed to assess the effects of ES on cell migration, as shown in Figure 5e. The results suggested that the groups receiving electrical treatment demonstrated remarkable migration, which was attributed to the enhanced electric field generated by the HSESES, thereby boosting the migration distance and speed of the cells [11, 13, 14, 52]. Specifically, cells in ES groups (BM, EM, and BM+EM) migrated 125%, 165%, and 270% faster, respectively, than those in the control groups over a 6 h period (Figure 5f).

Angiogenesis is crucial in wound‐related diseases [54, 55, 56]. Accordingly, the influence of ES on human umbilical vein endothelial cells (HUVECs) was thoroughly investigated. As shown in Figure 5g, ES‐treated groups exhibited significantly more extensive and well‐organized tubular networks than the control group. Notably, the BM+EM group demonstrated the most pronounced angiogenic response, with the highest density of branch points (62), the longest total tube length (68), and the highest expression level of angiogenic factors (375) (Figure 5h–j). These results could be attributed to ES‐induced modulation of endothelial cell behavior and angiogenesis‐related signaling pathways [57, 58, 59]. Collectively, these findings highlight the ability of ES to enhance endothelial cell functionality, which is critical for neovascularization, thereby accelerating wound healing.

**FIGURE 6:**
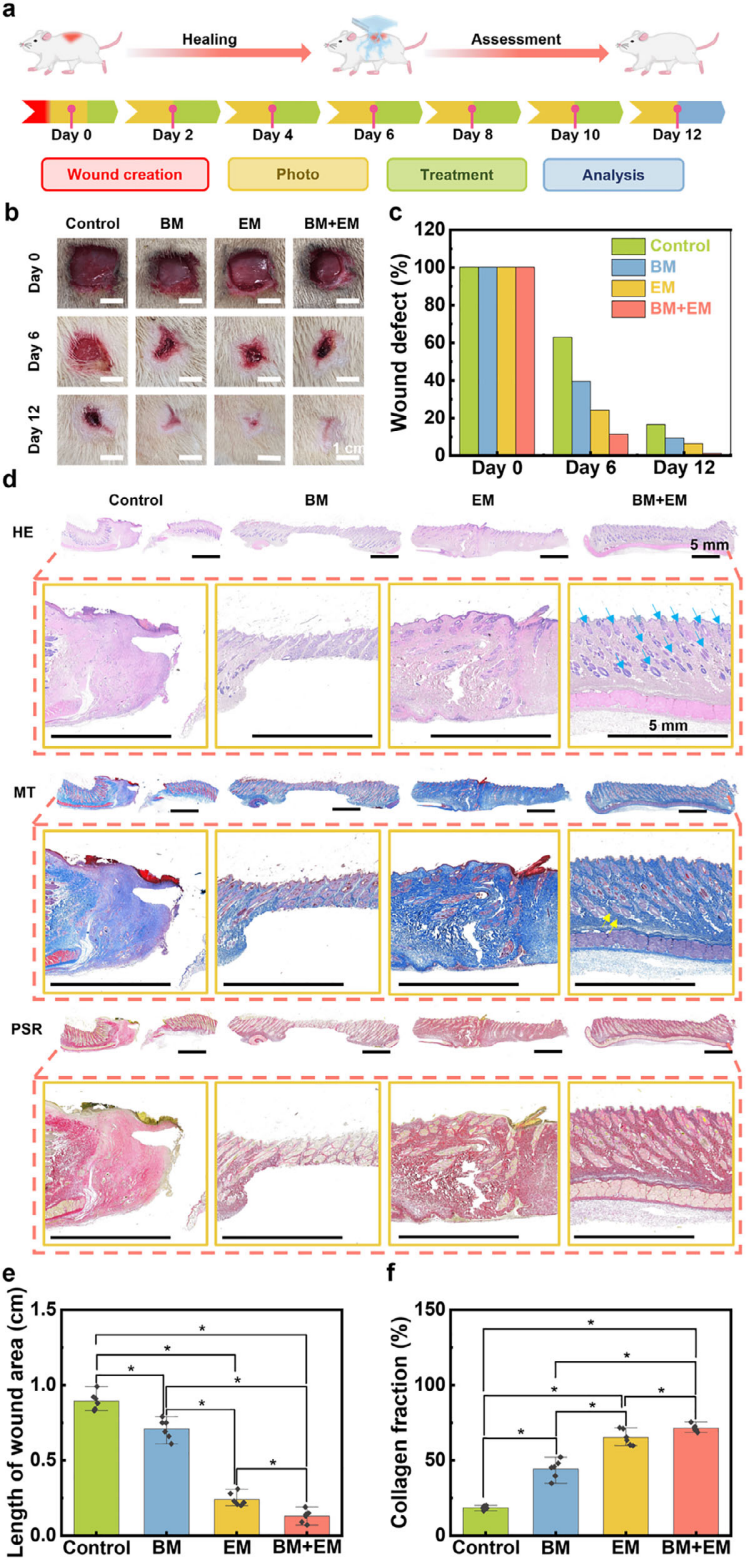
Responses of in vivo wound healing under ES. (a) Schematic of the HSESES applied to a full‐thick wound and a diagram of experimental procedures for wound‐healing therapy throughout the 12‐day period. (b) Representative photos of the wound‐healing process in each group. (c) Comparative evaluation of wound area progression across different treatment groups over an equivalent time course. (d) Representative images of HE, MT, and PSR staining of wounded tissue sections on day 12 following various treatments, along with corresponding higher‐magnification images (Blue and yellow arrows represent hair follicles and angiogenesis, respectively). (e) The wound area length and (f) collagen fraction after different treatments for 12 days.

To evaluate the effects of ES on wound healing, a full‐thickness skin wound model in rats was employed, and the healing process was systematically and comprehensively analyzed. The overall experimental procedure is schematically illustrated in Figure 6a. Rats were randomly assigned to four experimental groups: control, BM, EM, and the combined BM+EM groups. For the stimulation groups, ES was applied once every 2 days throughout the treatment period. Wound healing progression was tracked at specific time points by quantifying residual wound area. Figure 6b illustrates the wound healing progression in each experimental group over time. Notably, distinct differences in wound closure were observed as early as 6 days post‐treatment. By day 6, the control group retained approximately 62% of the original wound area, whereas the BM, EM, and BM+EM groups decreased to 39%, 24%, and 11%, respectively. By day 12, the BM+EM group showed near‐complete wound closure, whereas the control group exhibited a wound area of approximately 16.5%, further highlighting the therapeutic potential of this device for promoting tissue regeneration (Figure 6c). A comparison of previously reported gelatin‐ or GelMA‐based materials for wound healing is provided in Table S1.

To comprehensively evaluate ES‐mediated tissue regeneration, the wound‐healing process was examined at the histological and micro/molecular levels by the end of the 12‐day treatment period. Histological assessments were performed using hematoxylin and eosin (H&E), Masson's trichrome (MT), and picrosirius red (PSR) staining, as shown in Figure 6d. H&E staining provided a detailed visualization of epidermal regeneration and granulated tissue formation at the wound sites. The control group exhibited incomplete full‐thickness healing with a pronounced tissue gap. In contrast, all ES‐treated groups exhibited faster healing responses. In the BM group, re‐epithelialization was evident; however, the deeper dermal layer remained incompletely regenerated. The EM group exhibited epidermal and dermal repair; however, the dermal architecture remained disorganized, indicating incomplete remodeling. The BM+EM group demonstrated well‐organized regeneration in both layers. These effects could be attributed to ES generated from the combined BM and EM modes, which more effectively enhance cell proliferation and migration, thereby supporting efficient regeneration [60, 61]. Notably, in the BM+EM group, the presence of reconstructed skin appendages, including the microvasculature and neogenic hair follicles, was identified, providing clear evidence of a more comprehensive and functionally mature tissue restoration process. These findings were even more distinctly evident in the corresponding higher‐magnification H&E‐stained images, which further highlighted the differences in tissue architecture among the groups.

Quantitative wound length analysis further supported these findings. The control group exhibited only approximately 9% wound closure, whereas the BM, EM, and BM+EM groups reached 31%, 75%, and 90%, respectively (Figure 6e). MT staining revealed enhanced collagen deposition in the wound area. Collagen, a key component of tissue repair, enhances tissue elasticity and supports cell migration and proliferation [62, 63]. The BM+EM group exhibited a significant number of newly formed and well‐organized collagen fibers with a collagen deposition rate of up to 72%. In contrast, the control group only showed approximately 18% collagen deposition, indicating a limited healing effect (Figure 6f). Moreover, a substantial number of newly formed hair follicles were observed in the BM+EM group, indicating advanced tissue regeneration. These enhancements could be associated with ES‐induced cell migration, which is consistent with the in vitro migration results and PSR staining [21, 24].

**FIGURE 7:**
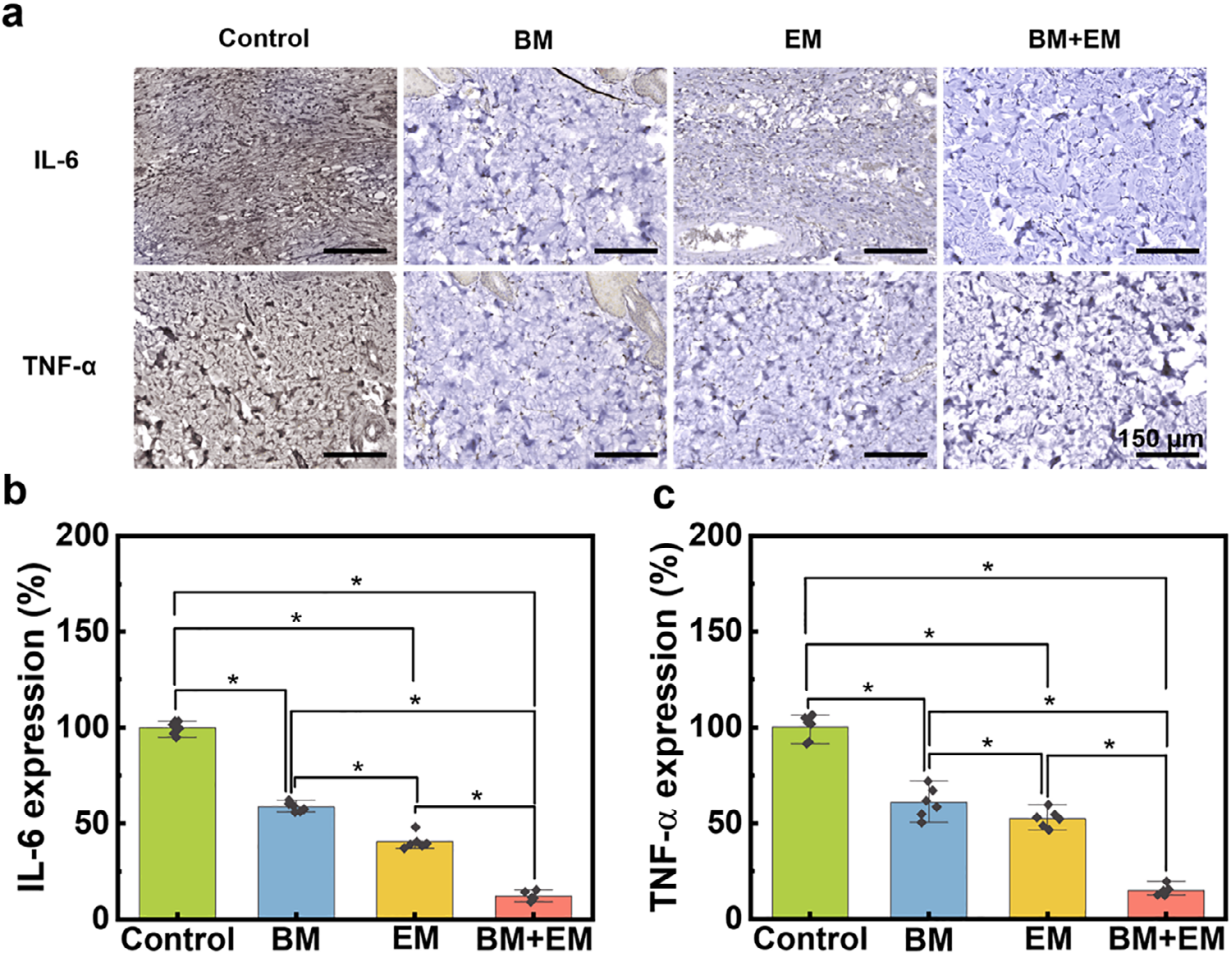
Evaluation of in vivo wound healing under ES. (a) IHC images of IL‐6 and TNF‐α. The corresponding enzyme‐linked immunosorbent assay (ELISA) results of (b) IL‐6 and (c) TNF‐α.

Immunofluorescence staining was performed to analyze the expression of key immune‐related factors associated with tissue repair. Tumor necrosis factor‐alpha (TNF‐α) and interleukin‐6 (IL‐6) are major pro‐inflammatory cytokines and widely recognized biomarkers of inflammatory response [64, 65]. To assess the impact of ES on local inflammation, immunofluorescence staining for TNF‐α and IL‐6 was performed on regenerated skin tissues collected on 12 days post‐treatment (Figure 7a–c). The results revealed that all ES‐treated groups exhibited significantly lower TNF‐α and IL‐6 expression levels compared to the control group, indicating a notable anti‐inflammatory effect. Notably, the BM+EM group showed the lowest TNF‐α (15.6%) and IL‐6 (11.4%) expression levels, underscoring the superior anti‐inflammatory efficacy achieved through the synergistic combination of BM and EM energy. Collectively, these findings suggest that the integrated stimulation with e‐skin effectively attenuates inflammatory responses and accelerates wound healing.

## 3 Conclusions

We report a multifunctional self‐powered bioelectronic e‐skin comprising gelatin‐based outer hydrogels and a zwitterionic conductive inner layer. By harvesting hybrid triboelectric energy (from BM energy) and EM energy (from ambient appliances), this e‐skin serves as a self‐powered electrical stimulator for accelerating wound healing. The e‐skin achieves outstanding energy harvesting performance, generating V_oc_ of 33.8 V and I_sc_ of 0.92 µA under BM actuation (3 N, 4 Hz), and V_oc_ of ±13 V and I_sc_ of ±0.65 µA when harvesting EM energy from an operating laptop. This e‐skin addresses the limitations of conventional TENGs for harvesting low‐frequency BM energy, thereby significantly expanding the applicability of self‐powered devices in biomedical applications. Through in vitro and in vivo experiments, we systematically evaluated the individual and synergistic effects of BM and EM energy‐based ES on tissue repair. Stimulation with the HSESES greatly accelerated wound closure, facilitated the regeneration of the epidermal and dermal layers, and enhanced the deposition and organization of collagen fibers. Furthermore, this hybrid stimulation strategy effectively reduced the expression of pro‐inflammatory cytokines TNF‐α and IL‐6, demonstrating a robust anti‐inflammatory response. These outcomes are consistent with those of the cellular‐level assays and collectively underscore the therapeutic promise of this dual‐mode bioenergy‐harvesting e‐skin. These results offer a non‐invasive strategy with potential biocompatibility for promoting tissue regeneration and represent significant advancements in next‐generation flexible bioelectronic therapies for wound care and regenerative medicine.

## 4 Experimental Section

### 4.1 Materials

Gelatin (CAS No. 9000708), methacrylic anhydride (MA, C_8_H_10_O_3_, CAS No. 760930), ammonium persulfate (APS, (NH_4_)_2_S_2_O_8_, CAS No. 7727540), [2‐(methacryloyloxy)ethyl]dimethyl‐(3‐sulfopropyl)ammonium hydroxide (SBMA, CAS No. 3637261), and sodium chloride (NaCl, CAS No. 7647145) were purchased from Sigma‐Aldrich (St. Louis, MO, USA). Human dermal fibroblasts (HDF) and the fibroblast medium were purchased from ScienCell Research Laboratories (Carlsbad, CA, USA).

### 4.2 Synthesis of Methacrylated Gelatin (Gel‐MA)

Gelatin powder (2.5 g) was dissolved in 25 mL deionized water at 40°C. Subsequently, MA (0.38 mL) was added dropwise to the above solution and stirred for 18 h at 75°C. The solution was dialyzed against deionized water using a dialysis membrane (MWCO = 12 kDa, Biokit Biotechnology Incorporation, Toufen City, Miaoli Country, Taiwan) for 5 days at 40°C, followed by lyophilization.

### 4.3 Synthesis of CSBMA

SBMA powder (5.58 g) was fully dissolved in 5 mL deionized water at 40°C. NaCl (0.58 g) was added to the above solution and stirred for 30 min at 40°C. Next, APS (0.024 g) was subsequently added for polymerization for 24 h at 70°C, obtaining the CSBMA.

### 4.4 Fabrication of the HSESES

A mold with a square groove (50 × 50 × 1 mm^3^) was fabricated from acrylic boards. Gel‐MA (1.5 g) was dissolved in deionized water under continuous stirring, followed by the addition of APS (0.03 g) into the above solution. The resulting solution was poured into the groove and heated to 60°C for the polymerization, forming the outer layers (PG). After formation, the product was extensively washed with deionized water to remove residual monomers and initiators. CSBMA was introduced into another groove to serve as the core layer. Finally, the polymerized outer layers and the CSBMA‐based core were assembled into a sandwich‐like structure. After formation, the product was extensively washed with deionized water to remove residual monomers and initiators.

### 4.5 Characterization

Fourier‐transform infrared spectroscopy (FTIR, PerkinElmer Spectrum 100, Waltham, Massachusetts, USA) was employed to identify the functional groups of materials with the spectral range of 400 and 4000 cm^−1^ under 32 scans. X‐ray photoelectron spectroscopy (XPS, Thermo Fisher Scientific, ESCALAB Xi+, UK) was used to investigate the elemental composition and chemical bonding states. A microforce testing system (Tytron 250, Eden Prairie, MN, USA) was used to perform the standard tensile strain–stress tests at a stretching rate of 0.1 mm s^−1^. Adhesion tests were performed according to standard testing protocols (ASTM D3330 for peeling test and ASTM D1002 for lap shear strength measurement). The conductivities, open‐circuit voltage (*V*_oc_), transferred charge (Q_tr_), and charging curves were measured using a Keithley 6514 electrometer. The short‐circuit current (*I*_sc_) was measured using a Stanford low‐noise current amplifier (SR570). All human body‐related experiments involving wearable devices on volunteers were conducted in compliance with relevant laws and institutional guidelines and were approved by National Chung Hsing University. Informed consent was obtained from all participants before enrollment in the study. The authors confirm that all human participants provided informed consent for the publication of the images in all figures.

### 4.6 Cell Proliferation and Viability under ES

Cell proliferation and viability under ES were evaluated using live/dead staining and the PrestoBlue assay. HDF cells were cultured in fibroblast medium for testing. The platinum electrodes were sterilized using irradiation with ultraviolet light, soaked in 75% ethanol, and then washed three times with PBS. For live/dead staining, the HDF cells were seeded into 48‐well plates at a density of 10^4^ cells per well and incubated for 1 day at 37°C and 5% CO_2_. Subsequently, two parallel platinum electrodes were vertically inserted into the culture medium, with one electrode connected to the HSESES and the other was grounded. The HSESES was employed to provide ES through three energy‐harvesting strategies: i) a contact force of 2 N (BM) at a constant frequency of 4 Hz is applied to HSESES to generate an output voltage of 20 V. ii) The HSESES is positioned on a laptop powered by a 60 Hz AC outlet to harvest EM energy and subsequently produce a stable electrical output of 13 V. iii) Both energy conversion modes (BM/EM energy) are utilized simultaneously. Each ES treatment was performed for 30 minutes. Subsequently, live/dead staining was performed on days 2 and 4 of culture. The assay dye was prepared by mixing 10 µL of Calcein‐AM, 5 µL of propidium iodide (PI), and 5 mL of PBS. The cell‐seeded substrates were washed twice with 1 mL PBS and incubated in the dye solution at 37°C for 15 minutes. After staining, the samples were imaged using an inverted fluorescence microscope (Eclipse TE2000‐U, Nikon, Japan). Note that the intracellular esterase activity in viable cells convert non‐fluorescent Calcein‐AM into green‐fluorescent calcein (excitation: 490 nm, emission: 515 nm), whereas PI penetrate the compromised membranes of non‐viable cells and bound to DNA, producing red fluorescence (excitation: 535 nm, emission: 617 nm). Briefly, green fluorescence indicates viable cells, and red fluorescence indicates dead cells. For PrestoBlue assay, the HDF cells were seeded into a 96‐well plate at 5000 cells per well and further incubated for 1 day at 37°C and 5% CO_2_. ES was performed as described in the previous section. Subsequently, the PrestoBlue assay was conducted on day 2 and 4 of culturing. Briefly, 10 µL of PrestoBlue reagent was added to 100 µL of culture medium in each well and incubated in the dark at 37 °C for 60 min. A spectrophotometer (Infinite Pro M200, Tecan, Männedorf, Switzerland) was used to measure the absorbance at 570 nm with a reference wavelength of 600 nm. All experiments were performed in sextuplicate and the average was recorded.

### 4.7 Cell Viability of HSESES

Cell viability was evaluated using the CCK‐8 assay (ISO 10993–5) and live/dead staining. Sample (100 mg) was incubated in 1 mL medium at 37°C for 24 h. Cells (5 × 10^3^ cells per well) were seeded in 96‐well plates and treated with fresh medium or extracts. After washing with PBS, cells were incubated with CCK‐8 solution at 37°C for 90 min. Cell viability was calculated as the ratio of the sample to the control optical density (*n* = 3). Live/dead staining was performed following the same procedure as described above.

### 4.8 Cell migration under ES

The culture‐insert 2 Well system (ibidi GmBH) was employed to evaluate cell migration. HDF cells were evenly seeded into two chambers at a density of 10^5^ cells per chamber. After a day incubation, the insert was carefully removed to generate a defined cell‐free gap. Initial images of the gap areas were captured under bright‐field illumination using a microscope (Olympus BX53, Melville, NY, USA). The cells were then subjected to various ES conditions for 30 min and allowed to migrate for 8 h. Migration analysis was performed using the ImageJ software to quantify the cell‐free area at each time point. All experiments were performed in sextuplicate and the average was recorded.

The migration rate was calculated as the percentage reduction in the gap area over 8 h as follows:

$$\begin{array}{ccc} Migration\;rate\;\left(\%\right) & = & [\left(Initial\;gap\;area-Final\;gap\;area\right)\;/\\ & & \times \;Initial\;gap\;area]\;\times \;100 \end{array}$$

### 4.9 In Vivo Wound Healing Assay

Healthy Sprague‐Dawley rats (7–8 weeks old) were used in this animal study. All animal procedures were performed in accordance with the guidelines of the Animal Research Ethics Committee of the Laboratory Animal Center of China Medical University (CMUIACUC‐2020‐352‐1). After anesthesia and hair removal, a full‐thickness excisional wound (1 cm × 1 cm) was made on the dorsal skin of each rat under deep anesthesia. 24 rats were randomly divided into four groups: i) control, ii) BM, iii) EM, and iv) BM+EM groups. ES was performed as described in the previous section. Wound closure was documented and analyzed using ImageJ on day 0, 6, and 12 post‐treatment. At the end of the treatment period, the rats were sacrificed, respectively. Skin samples were obtained for paraffin sectioning, and each sample was stained with hematoxylin & eosin (HE), Masson's trichrome (MT), and Picro‐Sirius Red (PSR).

### 4.10 Evaluation of Tissue Inflammation

Upon treatment completion, wound tissue samples were collected to assess inflammatory responses. The protein expression levels of interleukin‐6 (IL‐6) and tumor necrosis factor‐alpha (TNF‐𝛼) were quantitatively analyzed using enzyme‐linked immunosorbent assay (ELISA). In addition, immunohistochemical (IHC) staining was performed on tissue sections using antibodies against IL‐6 and TNF‐𝛼. The IHC results were quantified by calculating the mean optical density (IOD/Area) of the positively stained regions. All experiments were performed in sextuplicate and the average was recorded.

## Conflicts of Interest

The authors declare no conflicts of interest.

## Supporting information




**Supporting File 1**: advs75940‐sup‐0001‐SuppMat.docx.

**Supporting File 2**: advs75940‐sup‐0002‐MovieS1.mpg.

**Supporting File 3**: advs75940‐sup‐0003‐MovieS2.mpg.

**Supporting File 4**: advs75940‐sup‐0004‐MovieS3.mpg.

**Supporting File 5**: advs75940‐sup‐0005‐MovieS4.mpg.

**Supporting File 6**: advs75940‐sup‐0006‐MovieS5.mp4.

**Supporting File 7**: advs75940‐sup‐0007‐MovieS6.mp4.

## Data Availability

The data that support the findings of this study are available on request from the corresponding author. The data are not publicly available due to privacy or ethical restrictions.
