# Supplementary material for: Electrical‐Stimulation Hydrogel Electronic Skin for Sustainable Hybrid Biomechanical–Electromagnetic Energy Harvesting and Accelerating Wound Healing
Source: Adv Sci (Weinh). 2026 Jun 15:e75940. Online ahead of print. doi: 10.1002/advs.75940 (PMC13336485; doi:10.1002/advs.75940)
Supplement: Supplementary file 1 — Supporting File 1: advs75940‐sup‐0001‐SuppMat.docx. [file ADVS-9999-e75940-s003.docx]

Supporting Information

**Electrical-Stimulation Hydrogel Electronic Skin for Sustainable Hybrid Biomechanical–Electromagnetic Energy Harvesting and Accelerating Wound Healing**

Syun-Hong Chou, Ming-Han Lu, Wei-Chen Peng, Wei-Chun Yung, Pei-Yi Liu, Yu-Hsuan Yang, Yi-Lin Huang, Cheng-Hung Tsai, Ting-Yu Yang, Xuan Zhang, Zhi-Xian Yan, Ying-Chih Lai*, San-Yuan Chen*

S. H. Chou, P. Y. Liu, Y. H. Yang, T. Y. Yang, X. Zhang, Prof. S. Y. Chen

Department of Materials Science and Engineering,

National Yang Ming Chiao Tung University, Hsinchu 30010, Taiwan

Email: sanyuanchen@nycu.edu.tw

M. H. Lu, W. C. Peng, W. C. Yung, Y. L. Huang, C. H. Tsai, Z. X. Yan, Prof. Y. C. Lai

Department of Materials Science and Engineering,

National Chung Hsing University, Taichung City 402202, Taiwan

Email: yclai@nchu.edu.tw

Prof. Y. C. Lai

Innovation and Development Center of Sustainable Agriculture, i-Center for Advanced Science and Technology, National Chung Hsing University, Taichung City, 402202, Taiwan

Department of Physics, National Chung Hsing University, Taichung City, 402202, Taiwan

Prof. S. Y. Chen

Graduate Institute of Biomedical Sciences, China Medical University, Taichung, Taiwan

School of Dentistry, College of Dental Medicine, Kaohsiung Medical University, Kaohsiung, Taiwan.


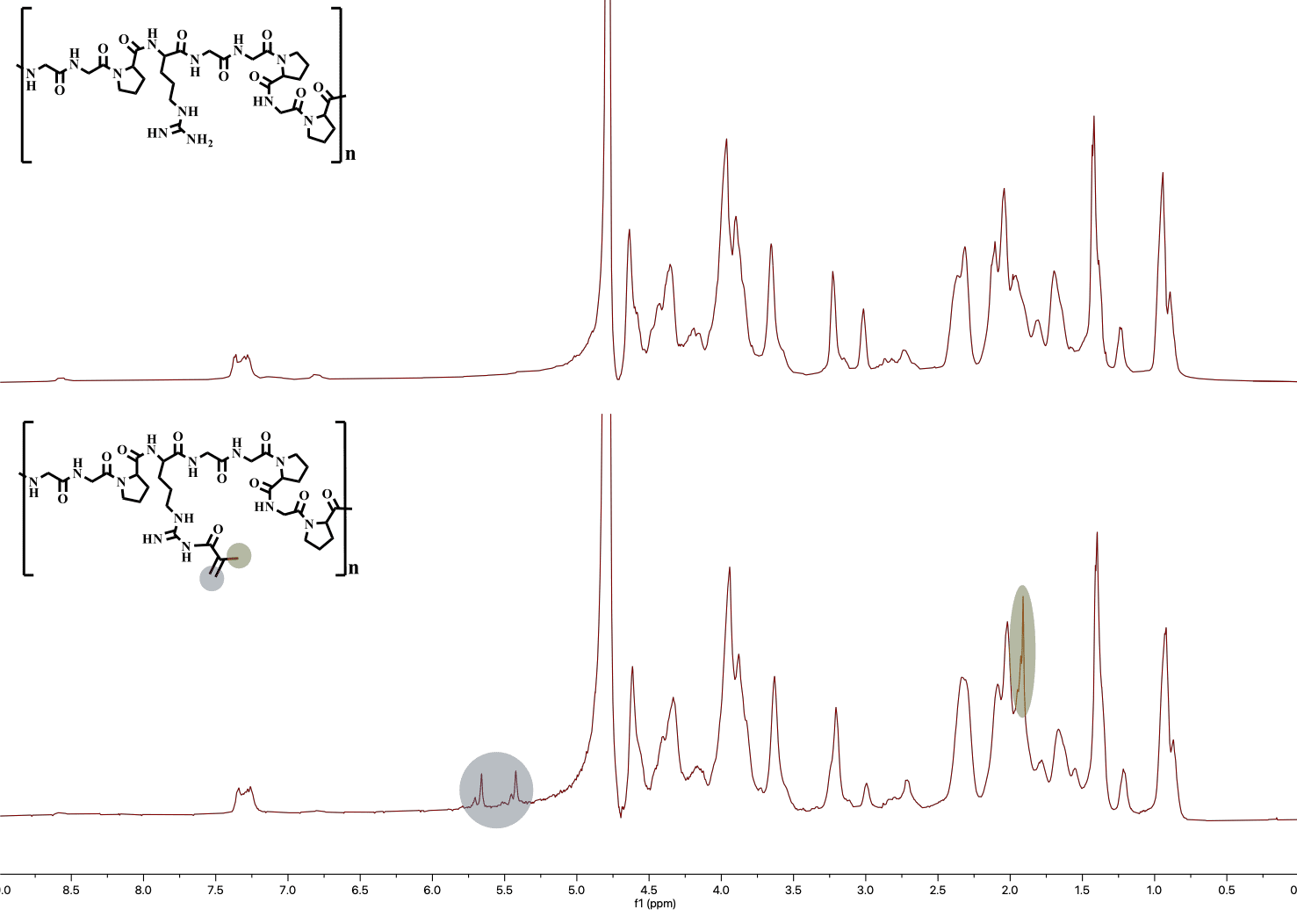


**Figure S1**. NMR spectrum of gelatin and Gel-MA. The new peaks at approximately 5.6 and 5.4 ppm in the spectrum indicate the presence of MA groups. Another new peak at 1.8 ppm is attributed to methyl protons of the MA groups. This evidence confirms the synthesis of Gel-MA.


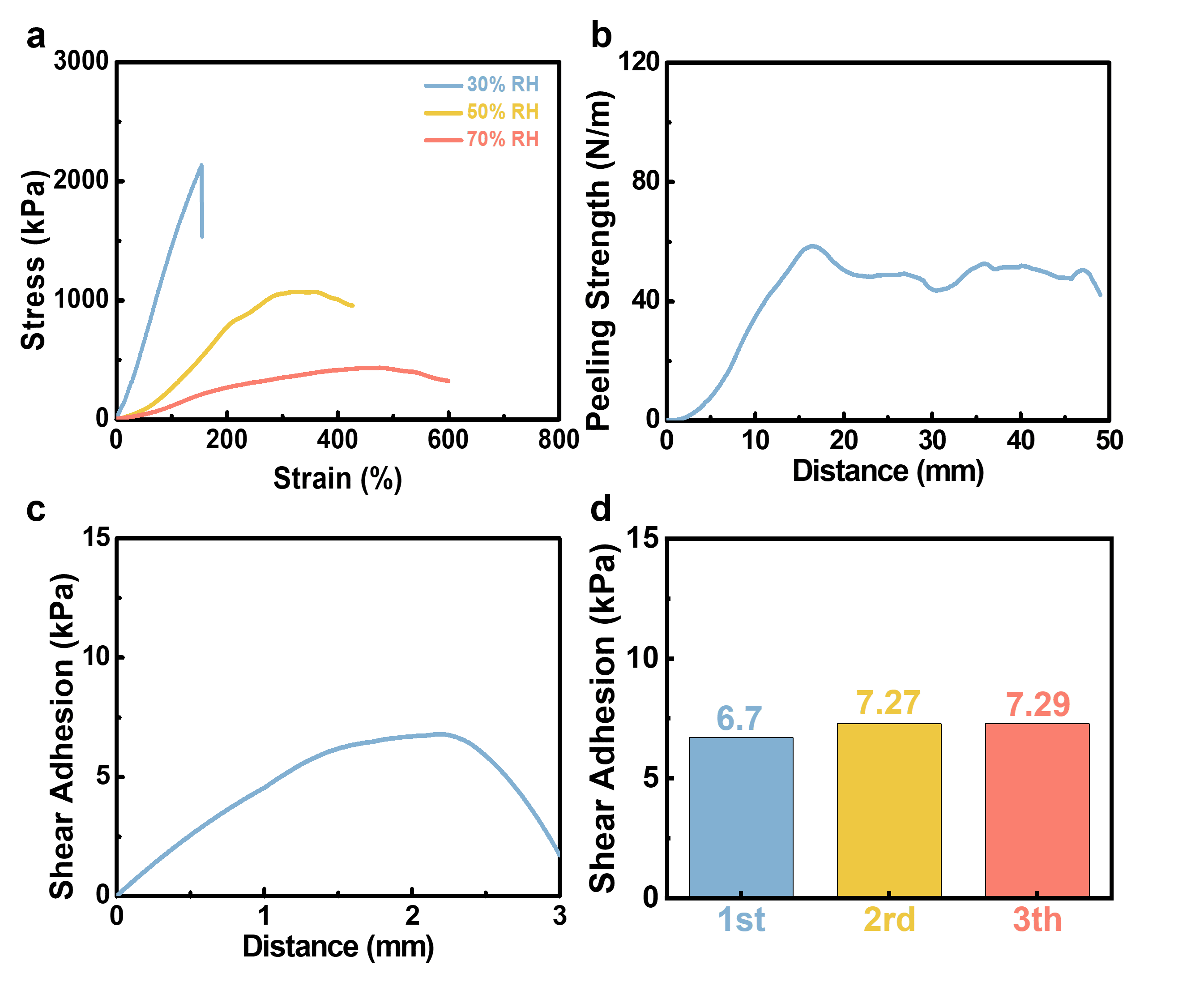


**Figure S2**. (a) Tensile stress-strain curves of the HSESES under various RH conditions. (b) Peeling strength-displacement curves of the HSESES. (c) Shear adhesion strength-displacement curves of the HSESES. (d) The cycle adhesion tests of the HSESES.


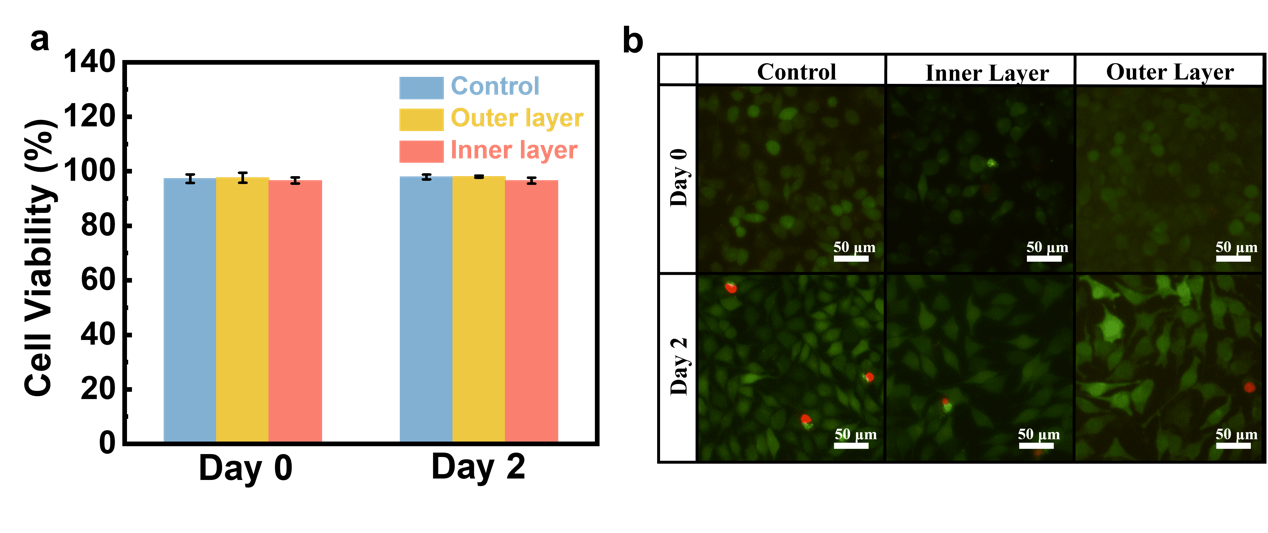


**Figure S3**. (a) Cell viability of cells cultured with the material extracts. (b) Live/dead fluorescence images of cells cultured with the material extracts. Note that n=3.


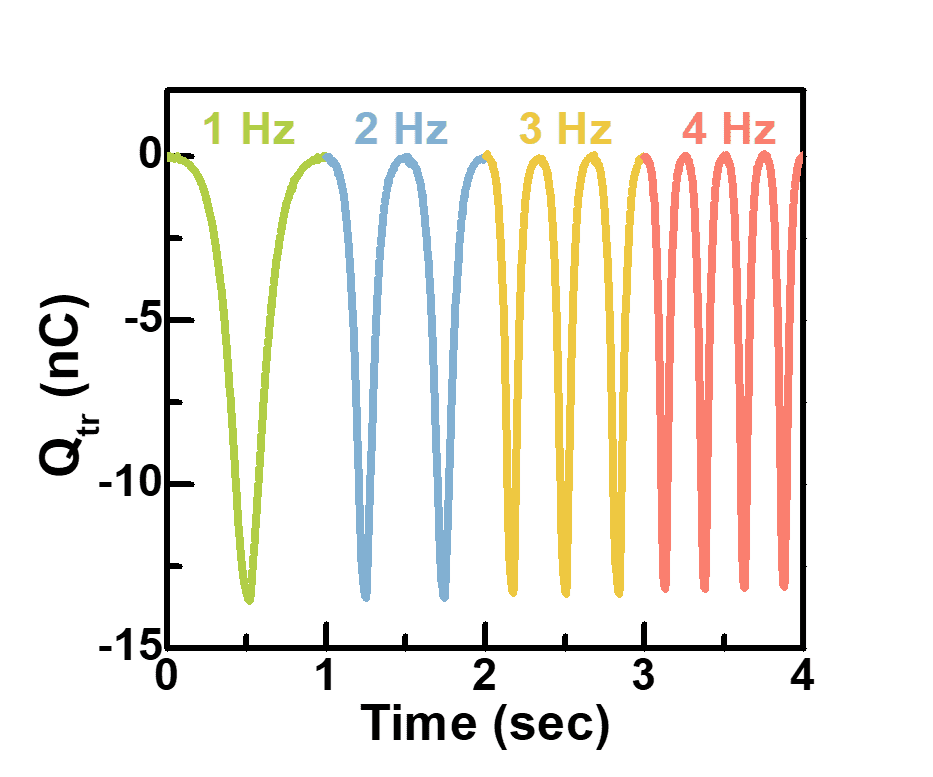


**Figure S4**. Outputs of Q_tr_ of the HSESES.


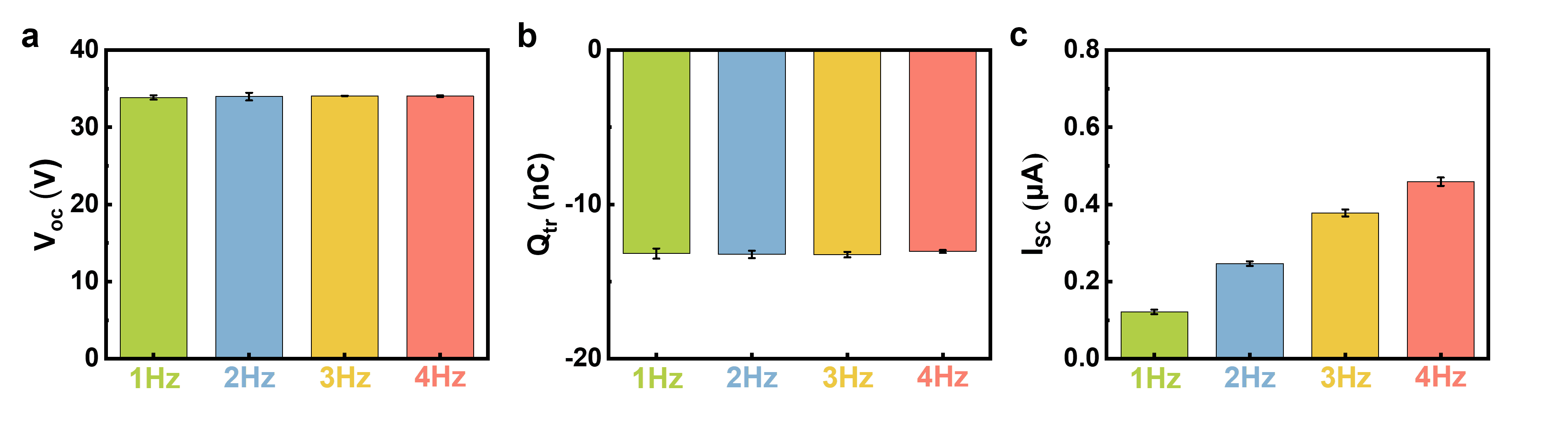


**Figure S5**. Outputs of (a) V_oc_, (b) Q_tr_, and (c) I_sc_ of the HSESES.


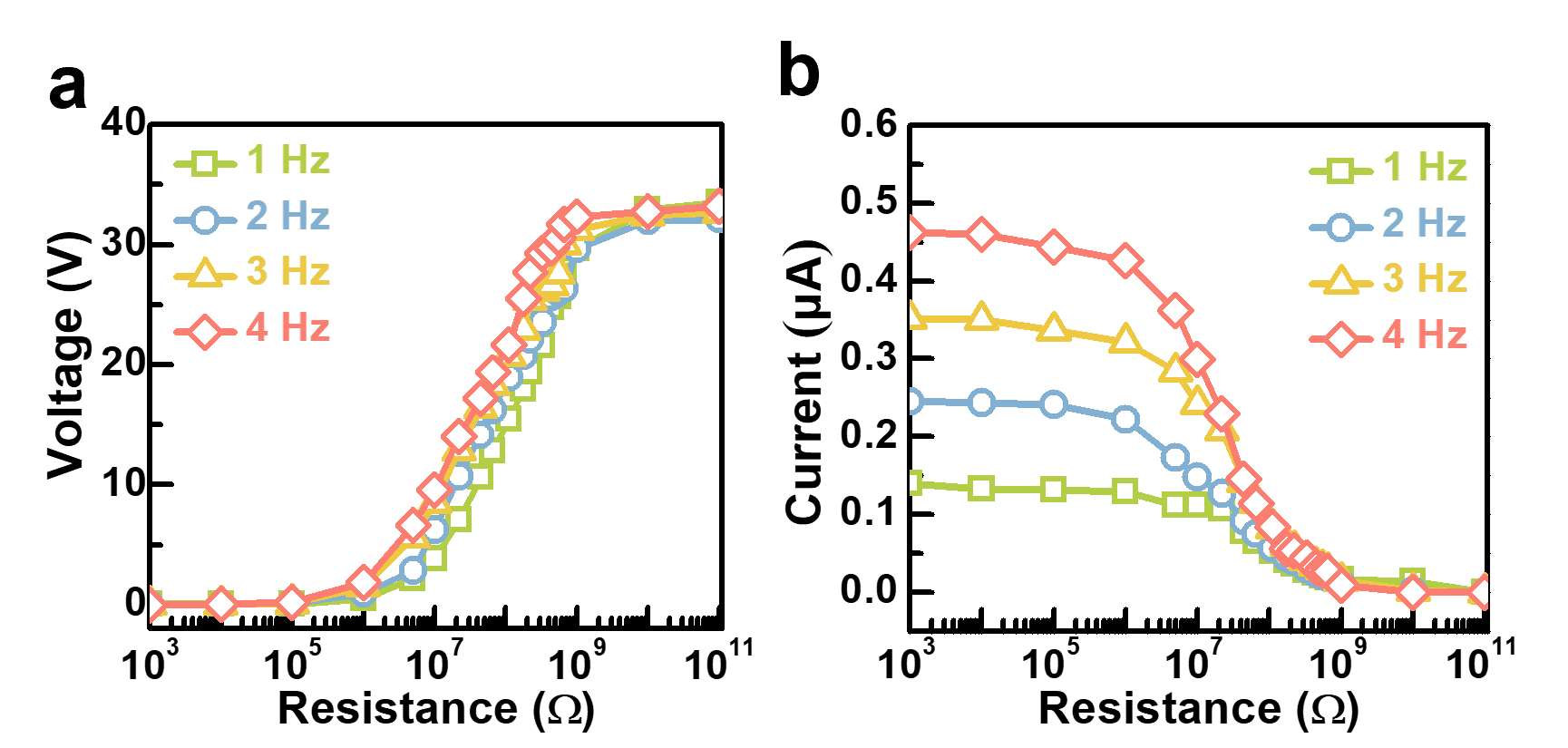


**Figure S6**. Relationships of outputs of (a) voltage and (b) current of the HSESES on different external load resistances.


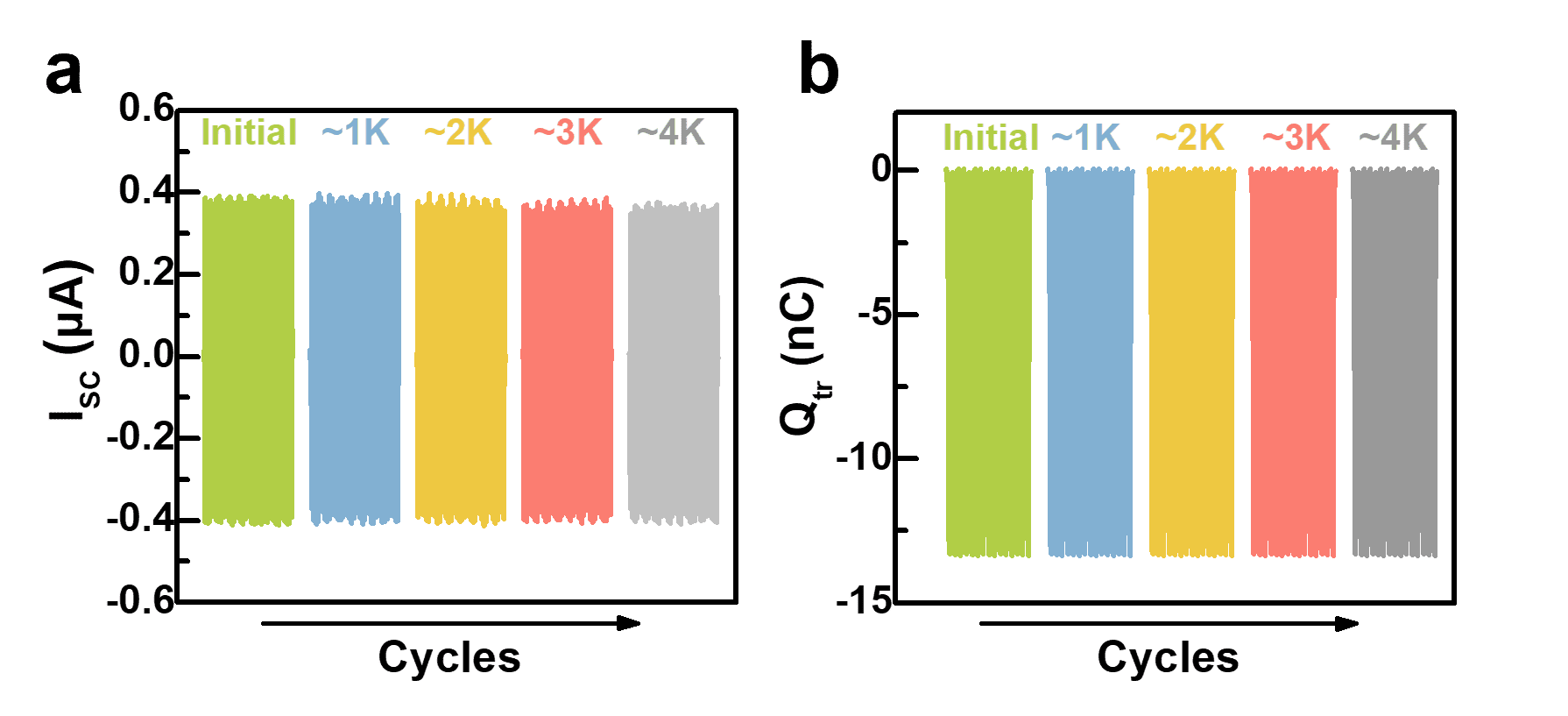


**Figure S7.** (a) Isc and (b) Q_tr_ of HSESES during a cyclic operation.


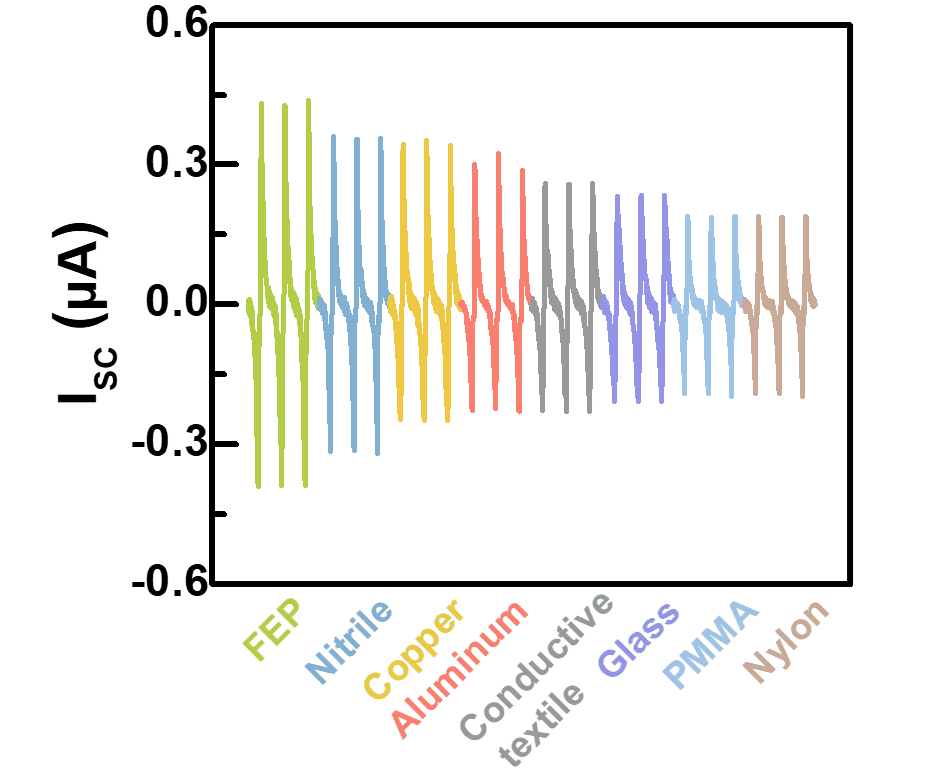


**Figure S8**. Isc of HSESES for various contact materials.


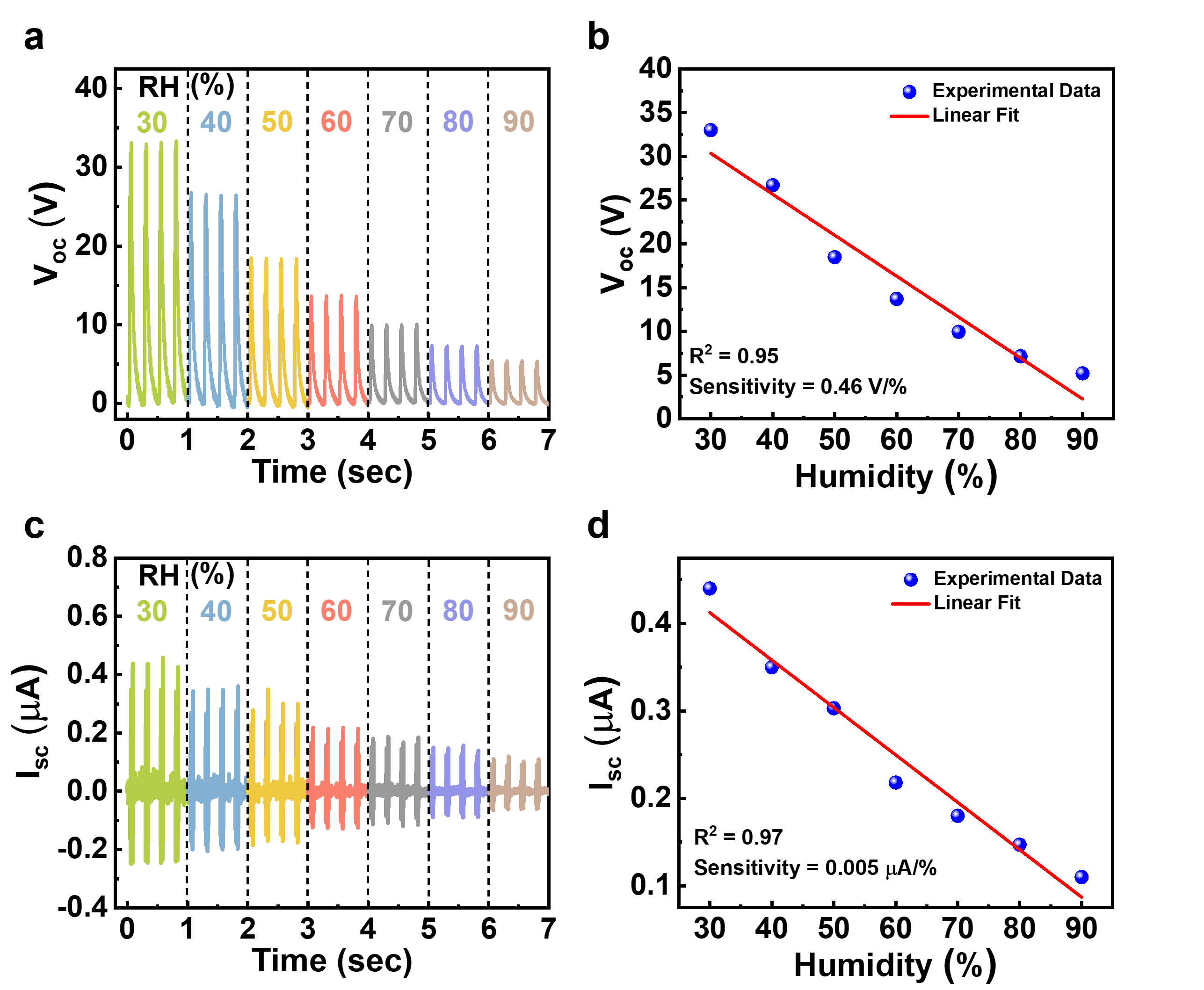


**Figure S9**. Real-time output (a) Voc and (c) Isc of the HSESES during mechanical energy harvesting under various humidity conditions and (b, d) the corresponding sensitivities.

The outputs of harvesting mechanical energy decreased from 33.8 to 5.41 V as the RH increased from 30% to 90%. Similarly, I_sc_ also decreased from 0.46 to 0.11 μA (Figure S9a and 9c). These results are attributed to charge dissipation induced by water molecules, indicating that elevated humidity significantly suppresses the output performances of harvesting mechanical energy.^[S1-S2]^ In addition, we also evaluated the linear relationship between the outputs and RH in Figure S9b and 9d. Both V and I exhibit high sensitivities of 0.46 V/RH% and 0.005 μA/RH%, respectively, indicating excellent responsiveness to humidity variations.


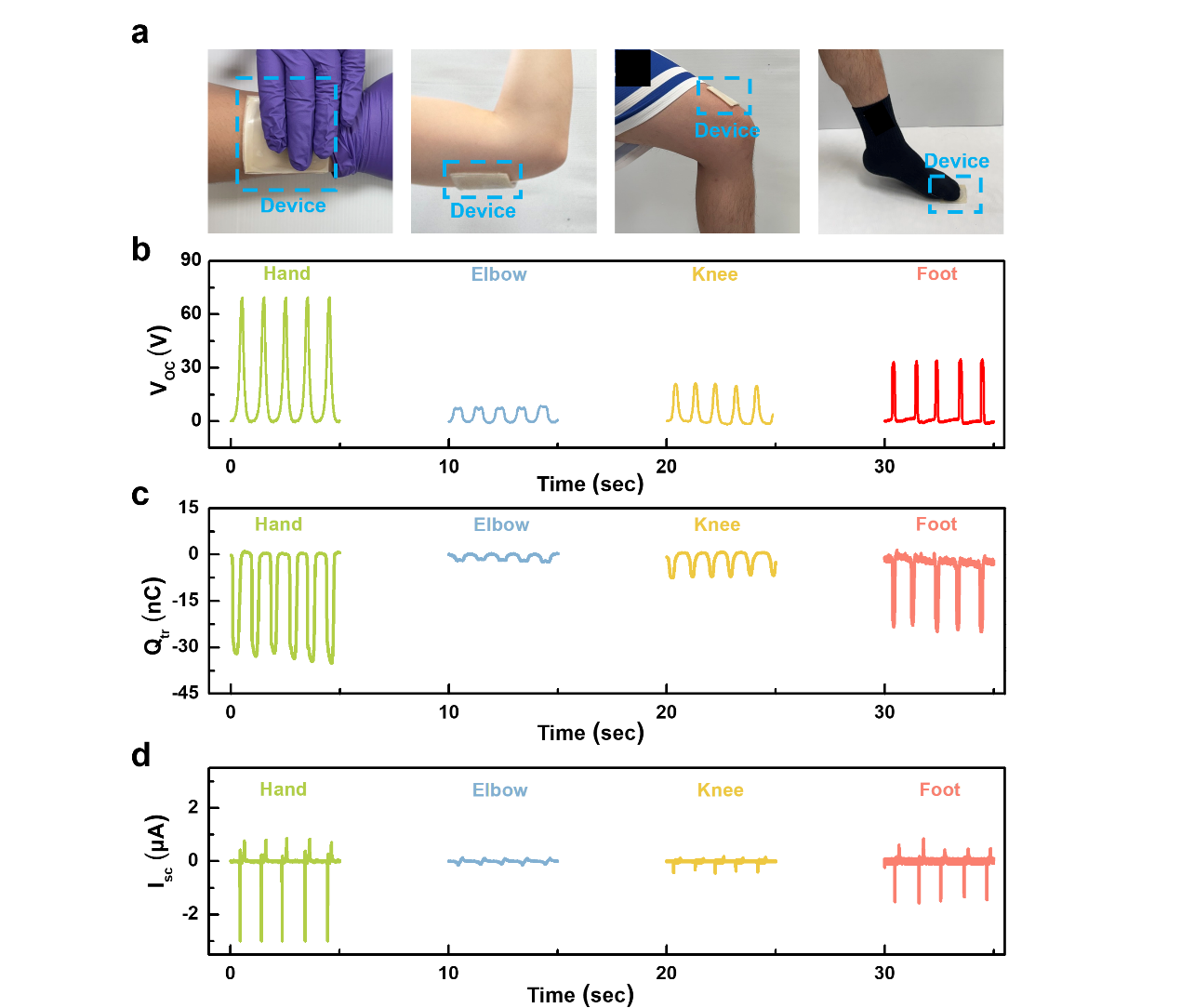


**Figure S10**. (a) Demonstration of use of the HSESES on different body parts. The corresponding outputs of (b) V_oc_, (c) Q_tr_, and (d) I_sc_.


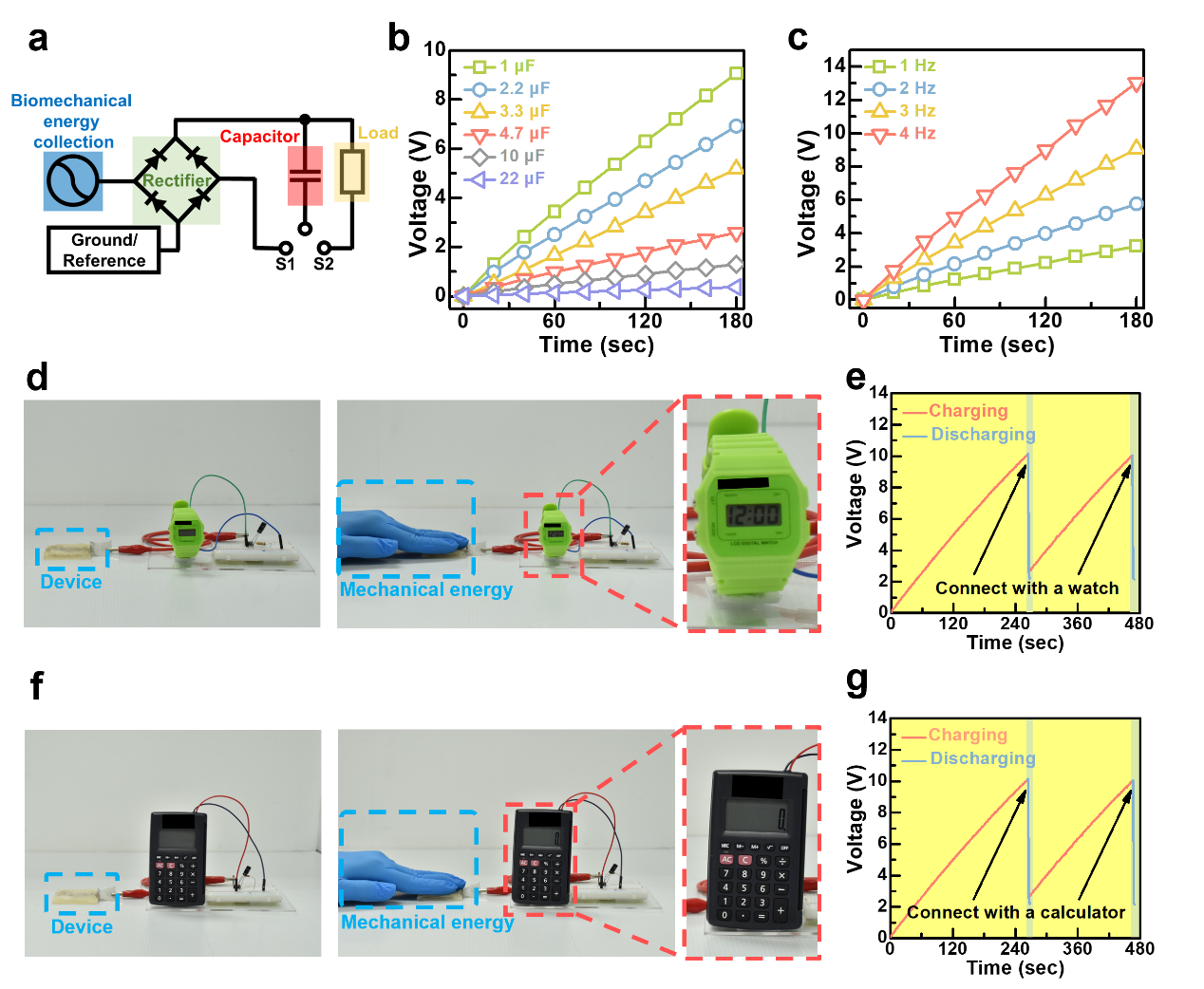


**Figure S11.** (a) Equivalent circuit for charging/discharging of the HSESES harvesting system. (b) Charging curves for various capacitors. (c) Charging curves for a 1 μF capacitor at various frequencies. (d) Photos of energy harvesting for powering an electronic watch, and (e) its real-time charging/discharging curves. (f) Photos of energy harvesting for powering a calculator, and (g) its real-time charging/discharging curves.

Figure S11b shows the distinct charging curves of the HSESES charging for capacitors with varying resistances under a 3N contact force and 3 Hz. For 180 seconds, the 1 and 22 μF capacitors were charged to 9 and 0.36 V, respectively, obeying the opposite tendency between the charging voltage and resistance. Moreover, the relationship between the charging behavior and charging speed is shown in Figure S11c. A 1 μF capacitor was charged to 3.2 and 13 V at the charging frequencies of 1 Hz and 4 Hz, respectively. The electricity produced by the HSESES was subsequently practically used to power an electronic watch and a calculator, as shown in Figure S11d, S11f, and Movie S1-2. The corresponding charging and discharging curves are shown in Figures S11e and S11g, respectively.


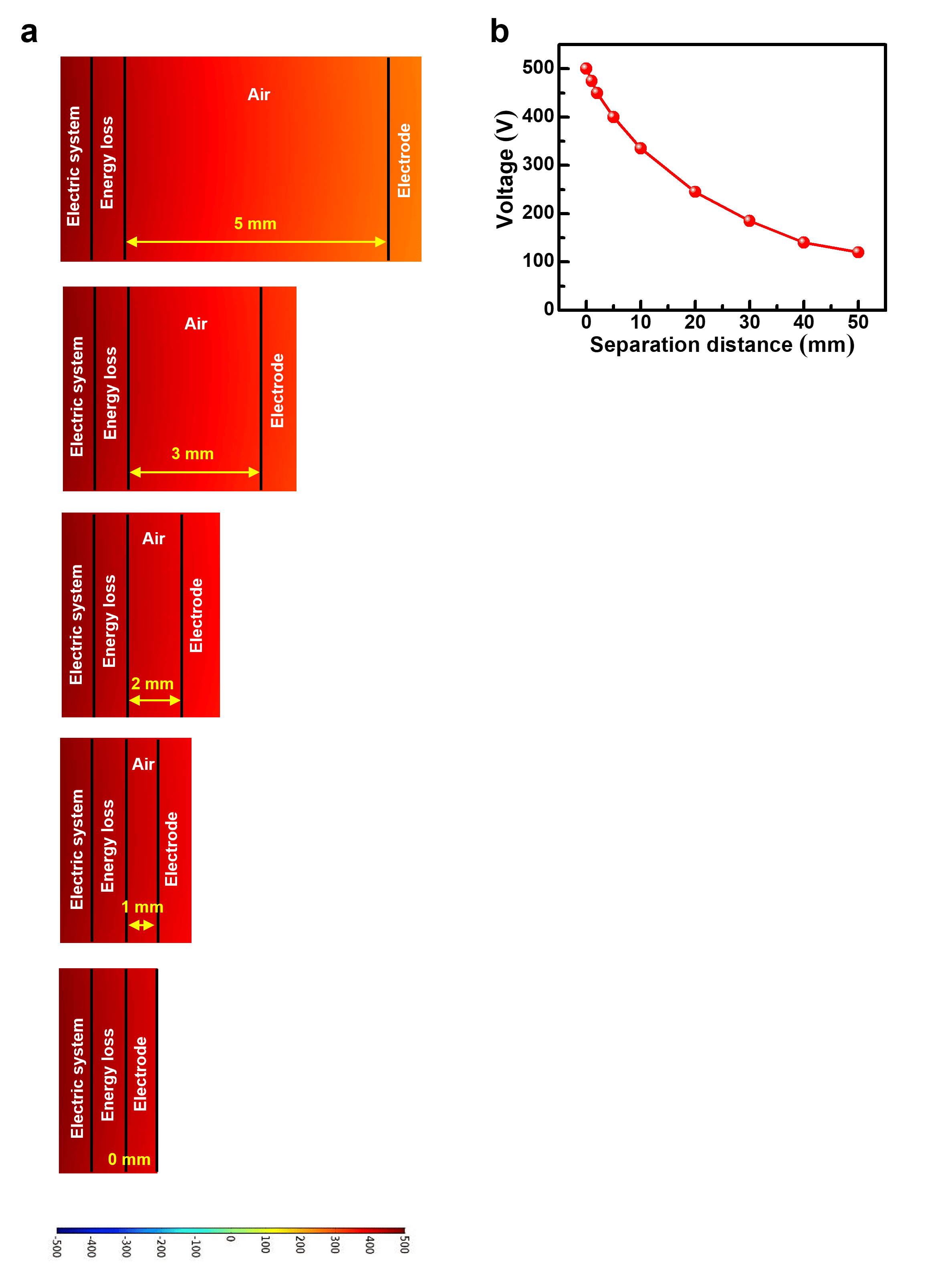


**Figure S12.** FEM electrostatic simulations of the HSESES. (a) Electric potential generated as the device approaches an external electric system. (b) Electrostatic potential distribution in the HTEH at varying distances from the electric system.


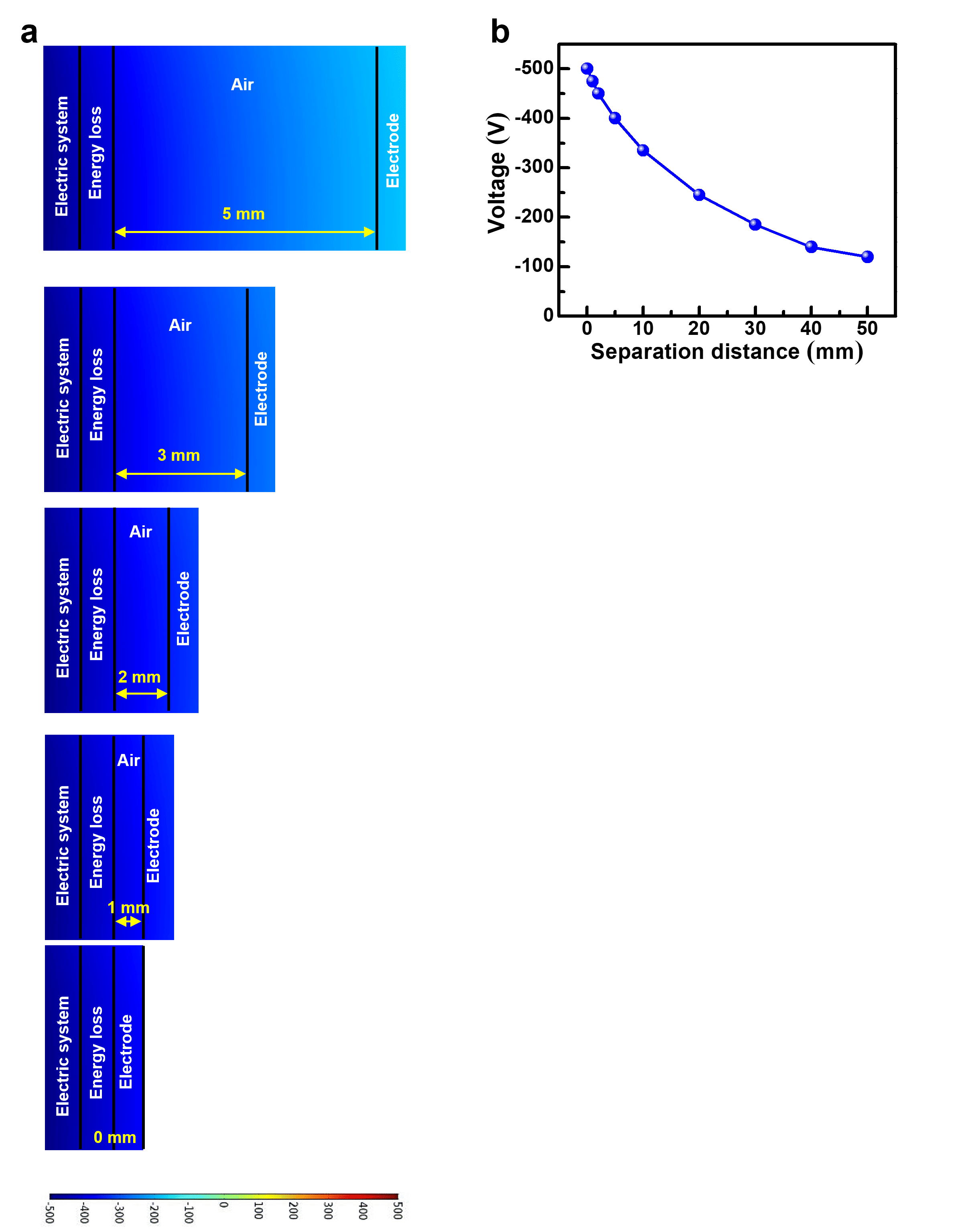


**Figure S13.** FEM electrostatic simulations of the HSESES. (a) Electric potential generated as the device approaches an external electric system. (b) Electrostatic potential distribution in the HTEH at varying distances from the electric system.


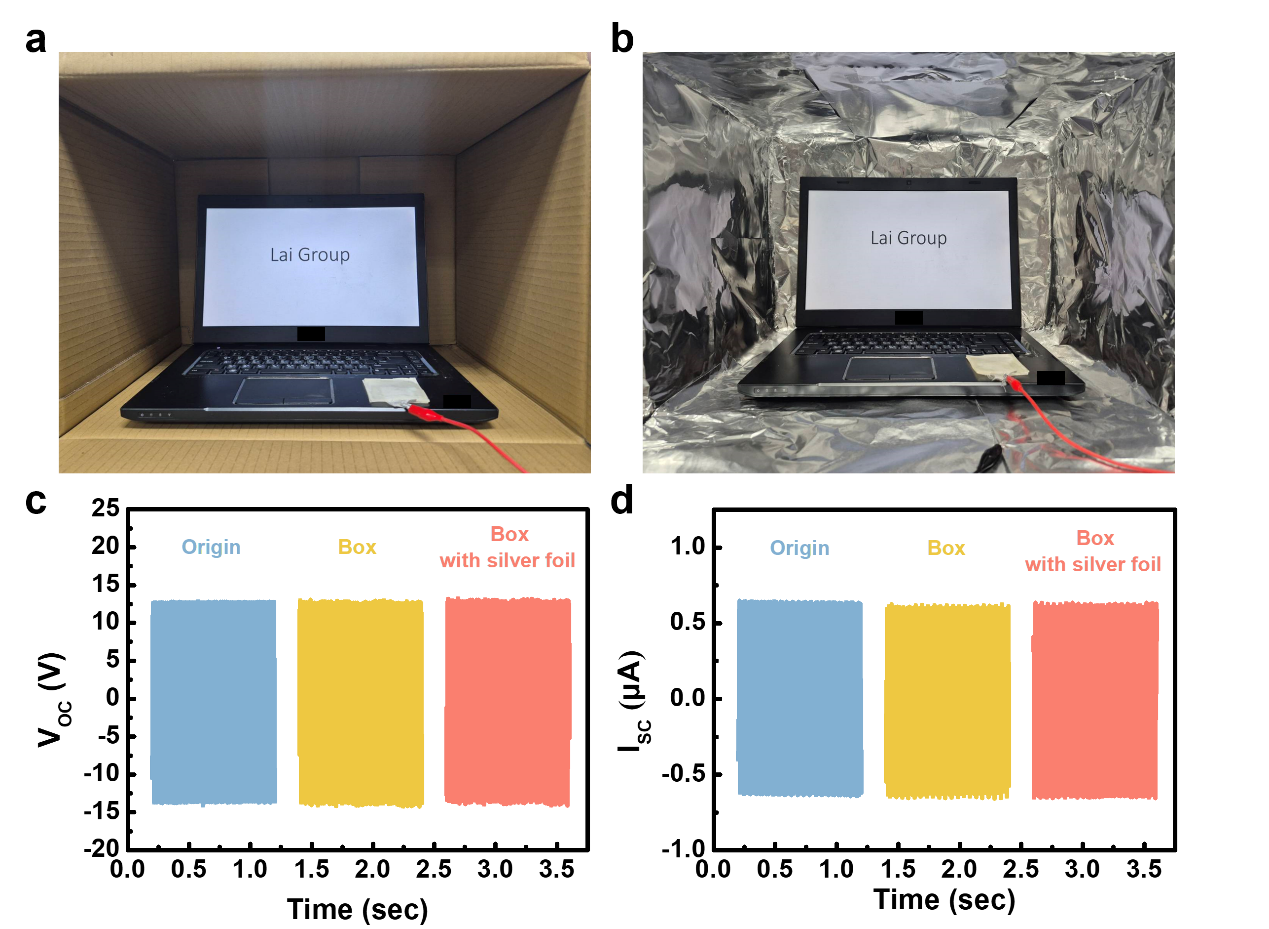


**Figure S14.** Images of the experimental setup (a) cardboard box and (b) Faraday cage. The corresponding outputs of (c) V_oc_ and (d) I_sc_.

Specifically, we designed two setup conditions to evaluate the influence of external environmental factors and electrostatic interference, as shown in Figure R15. In the first condition, the computer was placed inside a grounded cardboard box, and the device was positioned on the computer to harvest EM energy, enabling the comparison with measurements conducted under ambient conditions (Figure R15a). In the second condition, the computer was placed inside a cardboard box with conductive aluminum foil and grounded, and the device was positioned on the computer to harvest EM energy (Figure R15b). This design formed a Faraday cage to block external electric fields and eliminate internal electric fields. As observed in Figure R15c-d, the outputs of V_oc_ and I_sc_ exhibited no significant difference between ambient conditions and the cardboard-box one. In addition, under the Faraday cage condition, the output performance remained nearly constant. These results indicated that the measured outputs were primarily derived from the computer, with negligible influence from external interference.


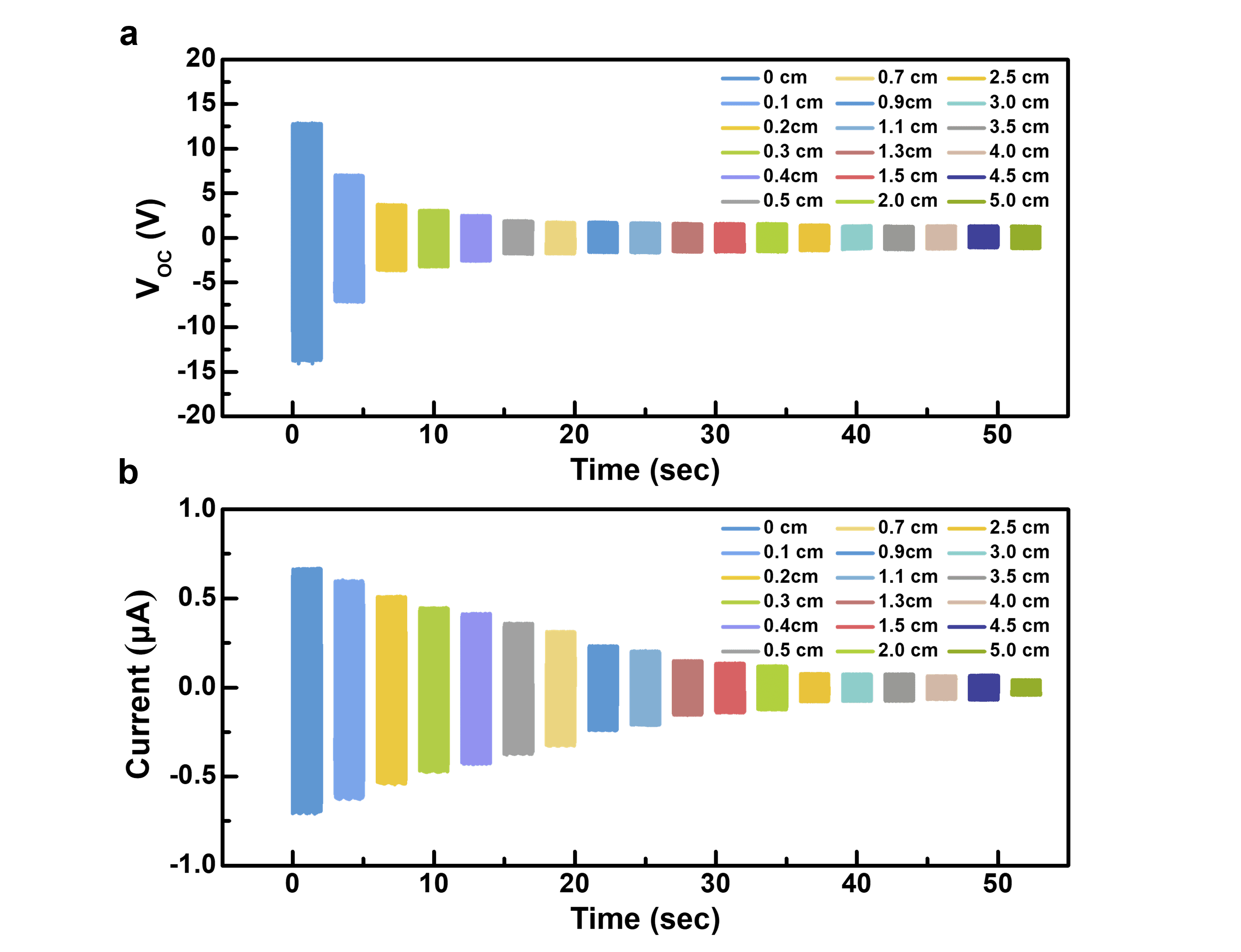


**Figure S15.** Dependence of (a) V_oc_ and (b) current on separation distances between the HSESES and a laptop.


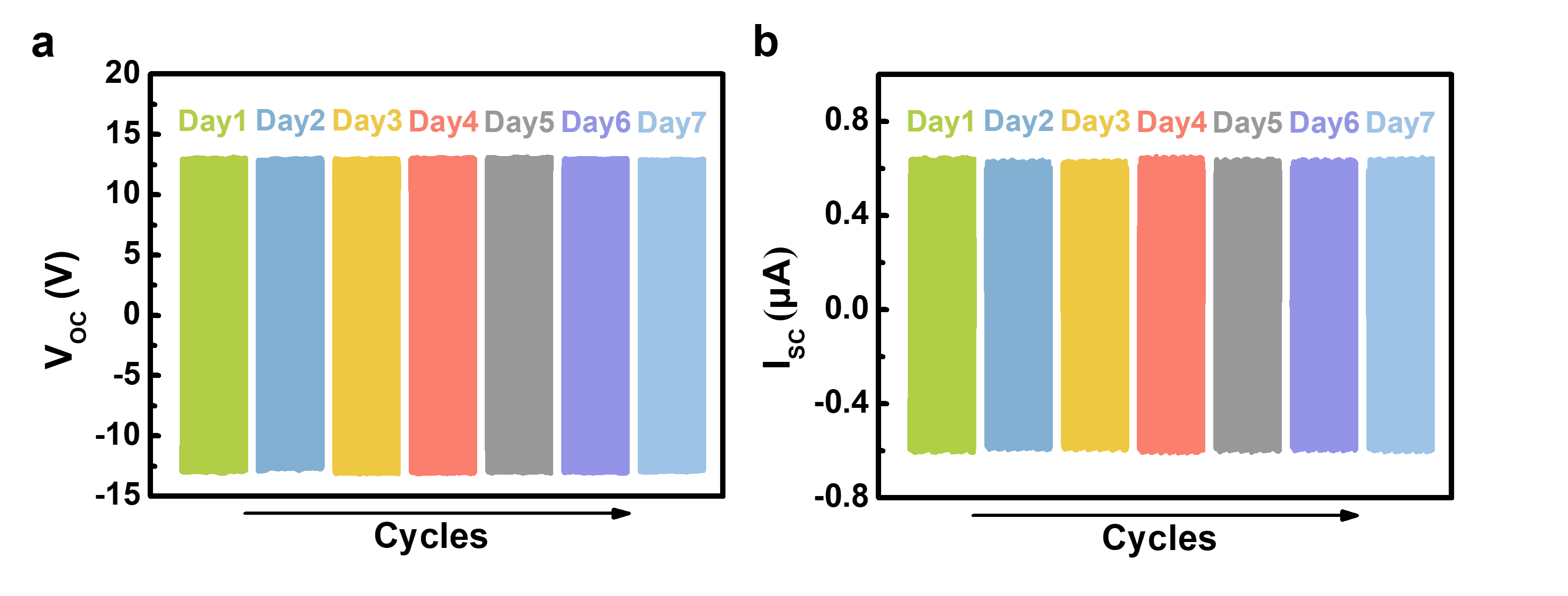


**Figure S16.** (a) V_oc_ and (b) I_sc_ during a 7-day operation.


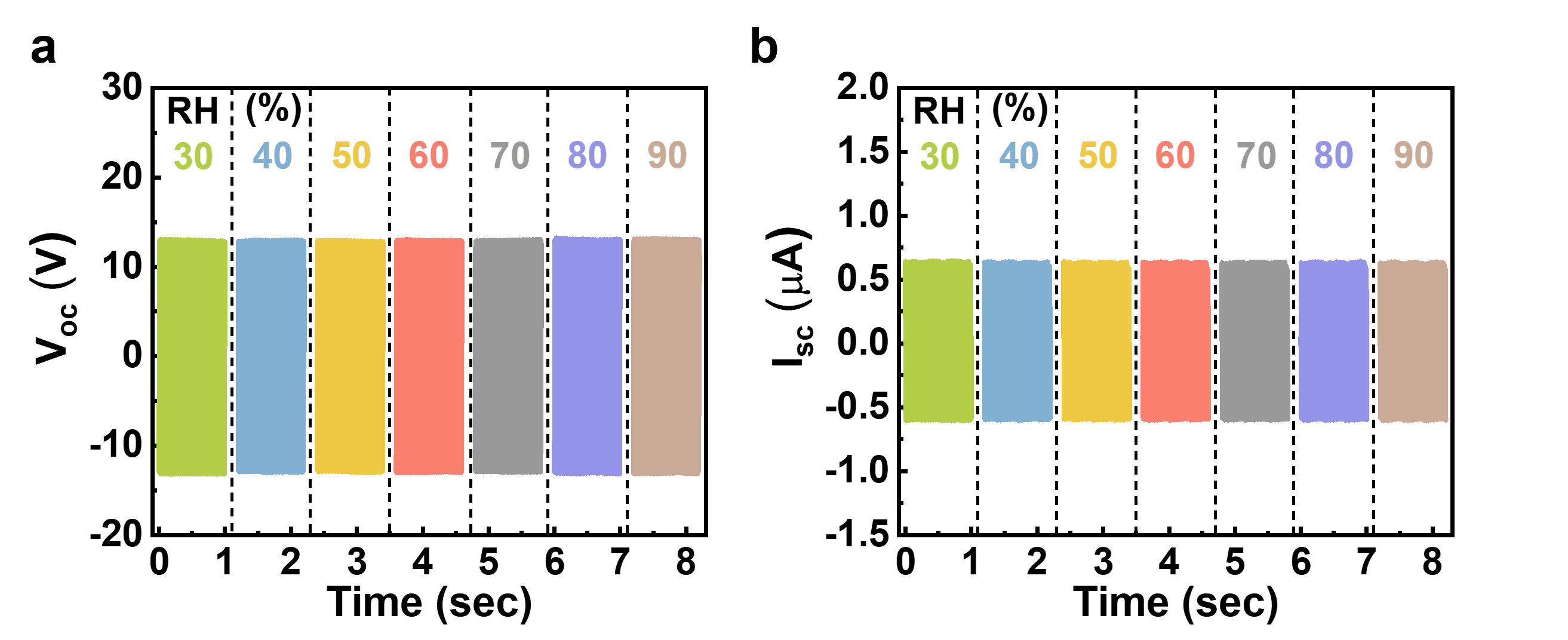


**Figure S17.** Real-time output (a) Voc and (b) Isc of the HSESES during EM energy harvesting under various humidity conditions.

The outputs of harvesting EM energy remained nearly constant for both V_oc_ and I_sc_ as the RH increased from 30% to 90%. These results are attributed to the EM energy harvesting mechanism, which relies on the induction of an alternating electrostatic potential in the conductive layer to capture dissipated EM energy.^[S3-S4]^ Notably, in our research, the conductive layer was effectively protected by the outer triboelectric layers, thereby minimizing the influence of humidity.


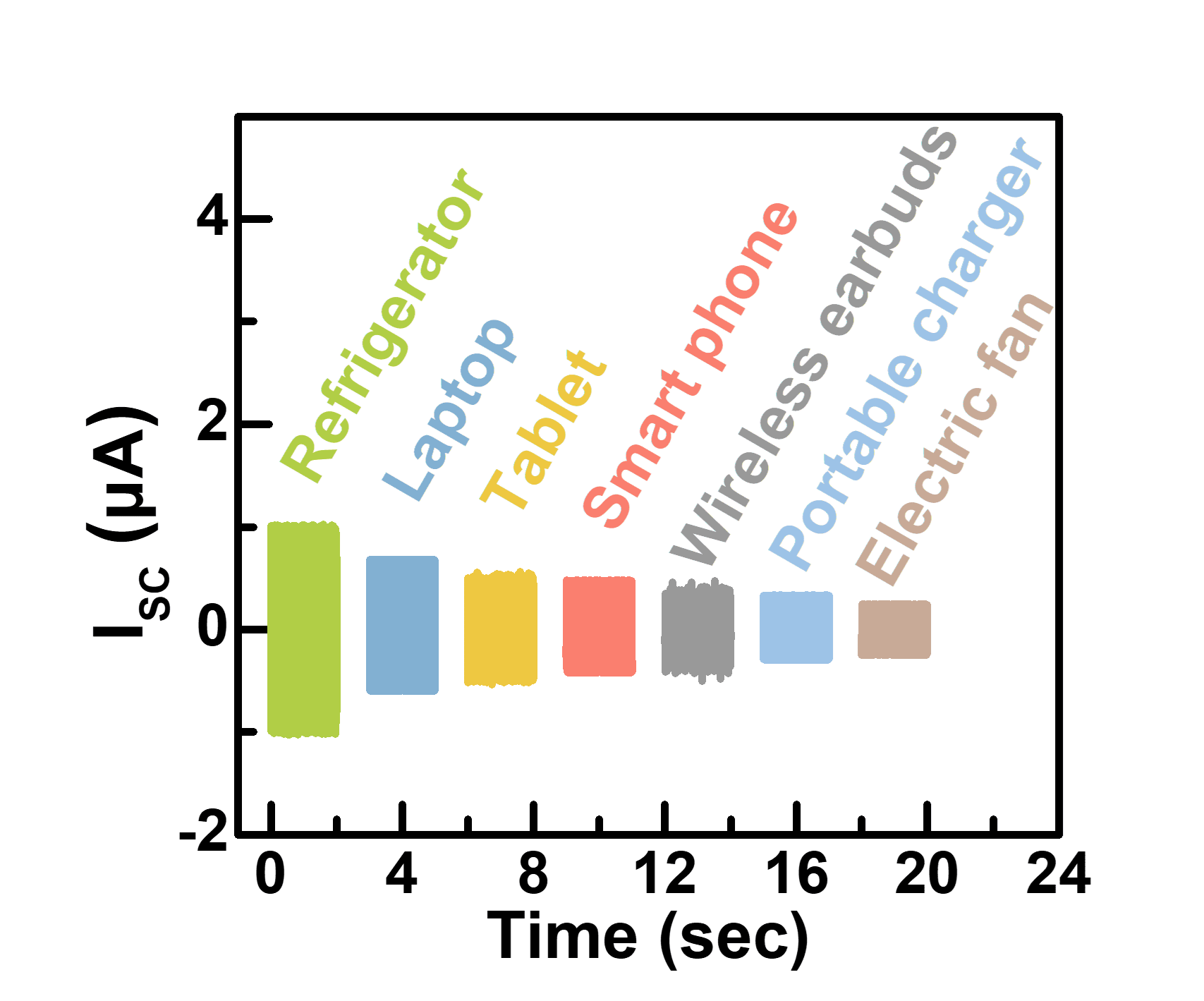


**Figure S18.** I_sc_ of scavenging EM energy dissipated from various electrical appliances using the HSESES.


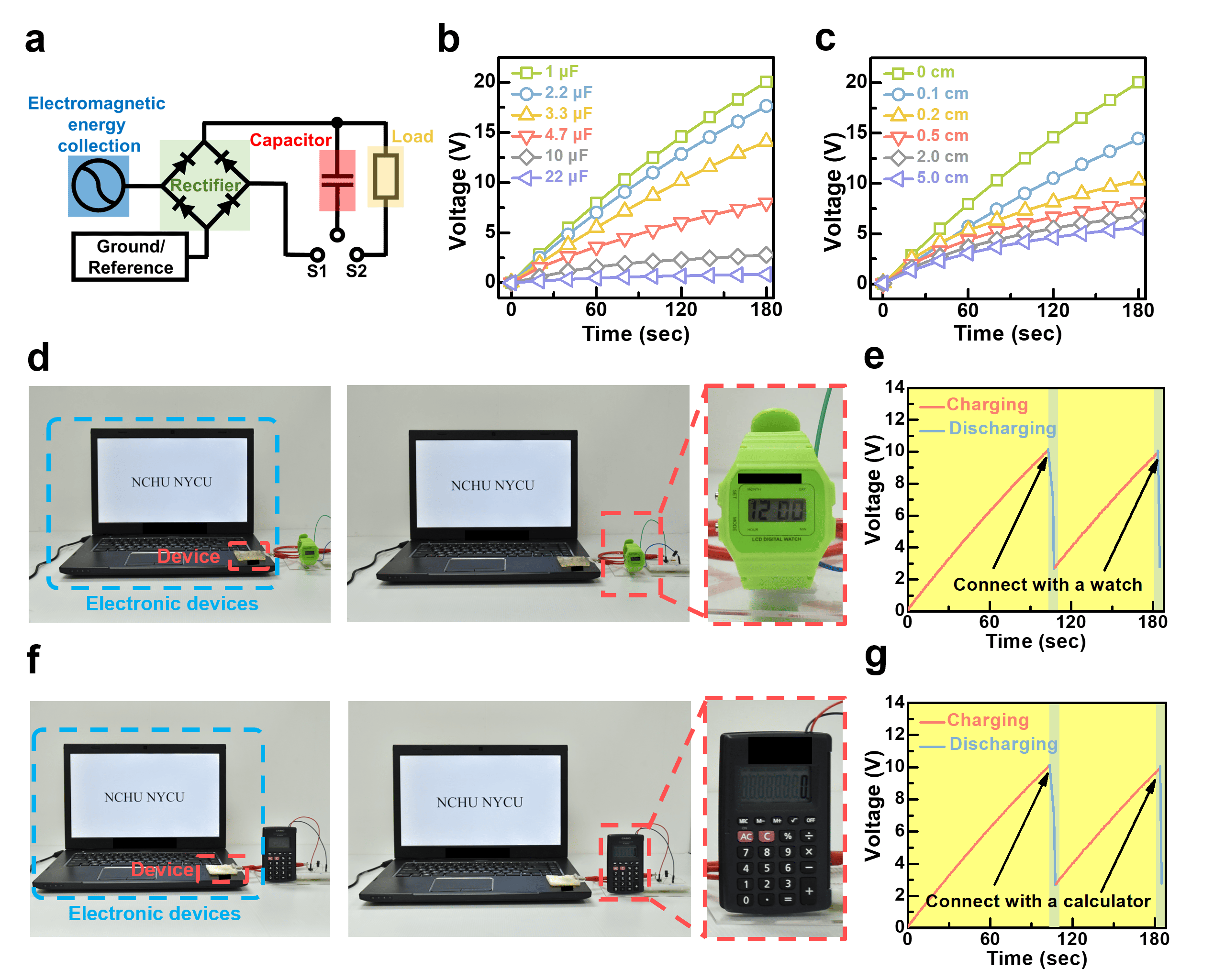


**Figure S19.** (a) Equivalent circuit for charging/discharging of the HSESES harvesting system. (b) Charging curves for various capacitors. (c) Non-contact charging curves for a 1 μF capacitor at different separation distances from 0 to 50 mm between the HSESES and a laptop. (d) Photos of scavenging EM energy from a laptop for powering an electronic watch, and (e) its real-time charging/discharging curves. (f) Photos of scavenging EM energy from a laptop for powering a calculator, and (g) its real-time charging/discharging curves.

Figure S19b displayed the distinct charging curves of the HSESES charging for capacitors with varying resistances while harvesting EM energy from a working laptop. After 180 seconds, the 1 and 22 μF capacitors were charged to 20 and 0.85 V, respectively. In addition, the HSESES was employed to charge a 1 μF capacitor at various distances of 0 and 5 cm between the HSESES and the working laptop, with the capacitor was charged to 20.0 and 5.6 V, respectively (Figure S19c). It demonstrates the effective wireless energy transfer capability of the HSESES and highlights its potential for contactless energy harvesting or self-powered electronic devices. Figure S19d and S19f illustrate the use of the HSESES to scavenge the EM energy to power an electronic watch and a calculator, respectively. The process is further visualized in Movie S3-4. The corresponding real-time charging/discharging curves are shown in Figure S19e and S19g, respectively.


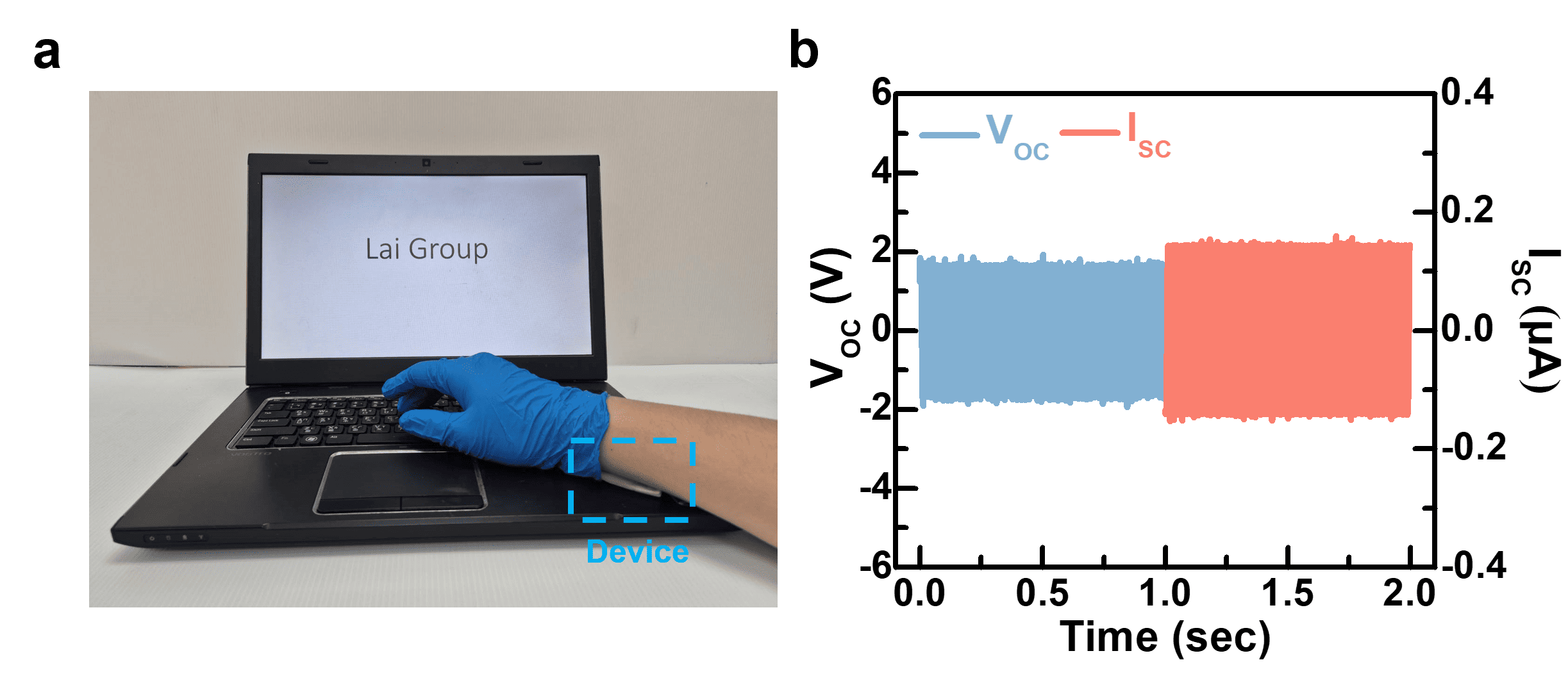


**Figure S20**. (a) Photos of EM harvesting during typing. (b) Relative outputs of V_oc_ (blue) and I_sc_ (red).


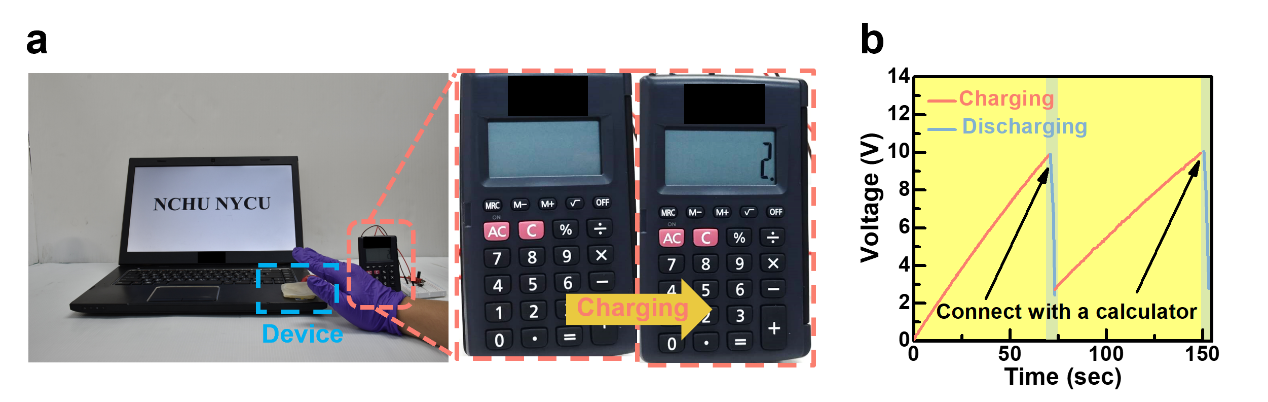


**Figure S21**. (a) Photos of harvesting hybrid energy for powering a calculator, and (b) its real-time charging/discharging curves.

| **Device materials** | **Adhesion** | **Stretch-ability** | **Output in harvesting BM energy** | **Output in harvesting EM energy** | **Treatment Strategy** | **Wound healing efficiency** | **Ref.** |
| --- | --- | --- | --- | --- | --- | --- | --- |
| **PG/CSBMA** | **Yes** | **150 %** | **33.8 V,**  **0.46 μA** | **±13 V,**  **±0.65 μA** | **Electrical stimulation**  **(Harvesting both BM and EM energy)** | **100 %,**  **12 day** | **This work** |
| Ecoflex/Gelatin methacryloyl/MXene | - | 450 % | 163.7 V,  8.1 μA | - | Electrical stimulation,  near-infrared photothermal effect | 99 %,  10 day | [S5] |
| Acrylamide/Stearyl methacrylate/Fish gelatin | Yes | 2600 % | 232 V,  1.6 μA | - | Electrical stimulation | 95 %,  10 day | [S6] |
| PTFE balls/PCB circuit board/Nylon films/Annular baffle/Yuhong ethanol extract/Gelatin methacryloyl | - | - | 142V,  0.62 μA | - | Electrical stimulation,  wound dressing | 100%,  12 day | [S7] |
| Hyaluronic acid methacryloyl/Gelatin methacryloyl/Graphene oxide quantum dots | Yes | - | - | - | Wound dressing | 90 %,  9 day | [S8] |
| Gelatin methacryloyl/ Ag₂Se | - | - | - | - | Thermoelectric effect | 100 %,  15 day | [S9] |
| Tannic acid-modified eggshell-based nanoparticles/ poly(lactic acid)/gelatin | - | - | - | - | Wound dressing | 95 %,  14 day | [S10] |
| MoS_2_/Gelatin methacryloyl/Ecoflex | - | 50 % | 42.48 V | - | Electrical stimulation,  near-infrared photothermal effect | 100 %,  10 day | [S11] |
| 4-(4-(hydroxymethyl)-2-methoxy-5-nitrophenoxy) butyrylethylenediamine-modified methacrylated gelatin/Epigallocatechin gallate-grafted polylysine/Resveratrol | Yes | 80 % | - | - | Wound dressing | 100 %,  14 day | [S12] |
| Cationic antibacterial agent ε-polylysine/Epigallocatechin-3-gallate/Methacrylated gelatin | - | 75 % | - | - | Wound dressing | 100 %,  14 day | [S13] |
| Gelatin methacryloyl/TEMPO-oxidized cellulose nanofibers/Zinc ions | Yes | 90 % | - | - | Wound dressing | 60 %,  14 day | [S14] |
| Gelatin-methacryloyl/Poly(3,4-ethylenedioxythiophene):poly(styrene sulfonate)/[2-(methacryloyloxy)ethyl]dimethyl-(3-sulfopropyl) | Yes | 501 % | - | - | Wound dressing | 97 %,  14 day | [S15] |
| Gelatin methacryloyl/Dopamine/Cerium oxide nanoparticles/Antimicrobial peptide | Yes | - | - | - | Wound dressing | 100 %,  14 day | [S16] |
| Gelatin methacrylate/Oxidized dextran/amino-modified poly(lactic-co-glycolic acid) | - | 100 % | - | - | Wound dressing | 99 %,  14 day | [S17] |
| Gelatin methacrylate/AgNWs | - | 206 % | - | - | Electrical stimulation,  wound dressing | 99 %,  10 day | [S18] |
| Tannin@ZnO microparticles/Gelatin methacryloyl/Polyaniline/PEGDA/2-Hydroxypropyltrimethyl ammonium chloride chitosan/ Hyaluronic acid | - | - | 9.64 V,  25.21 nA | - | Electrical stimulation | 97 %,  15 day | [S19] |

Table S1**.** Effects of gelatin/Gel-MA based materials on wound healing under different strategies

[S1] J. Zhang, C. Boyer, Y. Zhang, *Small* **2024**, 20, 2401846.

[S2] V. Nguyen, R. Zhu, R. Yang, *Nano Energy* **2015**, 14, 49.

[S3] S. Ginnaram, Y.-T. Chen, Y.-C. Lai, *Nano Energy* **2022**, 95, 107035.

[S4] Y. C. Lai, S. Ginnaram, S. P. Lin, F. C. Hsu, T. C. Lu, M. H. Lu, *Adv. Funct. Mater.* **2024**, 34, 2312443.

[S5] M. Mao, J. Kong, X. Ge, Y. Sun, H. Yu, J. Liu, W. Huang, D. Y. Wang, Y. Wang, *Chem. Eng. J.* **2024**, 482, 148949.

[S6] R. Yan, Q. Sun, X. Shi, Z. Sun, S. Tan, B. Tang, W. Chen, F. Liang, H.-D. Yu, W. Huang, *Nano Energy* **2023**, 118, 108932.

[S7] H. Liu, M. Zhang, L. Zu, J. Wen, H. Li, F. Xing, M. Yan, Z. L. Wang, B. Chen, *Nano Energy* **2024**, 125, 109585.

[S8] L. Wang, L. Sun, F. Bian, Y. Wang, Y. Zhao, *ACS nano* **2022**, 16, 2640.

[S9] Y. Qin, S. Jia, X.-L. Shi, S. Gao, J. Zhao, H. Ma, Y. Wei, Q. Huang, L. Yang, Z.-G. Chen, *Acs Nano* **2025**, 19, 15924.

[S10] B. Shao, Z. Mu, Z. Yuan, M. Shafiq, Z. Lei, H. Feng, L. Han, M. EL‐Newehy, M. M. Abdulhameed, J. Li, *Adv. Healthcare Mater.* **2025**, 14, e02979.

[S11] H. Yu, J. Kong, M. Mao, X. Ge, Y. Sun, J. Liu, J. Ye, Y. Wang, *Nano Energy* **2024**, 121, 109225.

[S12] X. Cao, Y. Deng, Z. Xu, T. Wang, B. Tang, J. Han, R. Guo, R. Yin, *Biofabrication* **2025**, 17, 015017.

[S13] X. Chen, J. Tang, Y. Dong, M. Xuan, Y. Tian, Y. Liu, N. Peng, B. Cheng, *Colloids Surf. B Biointerfaces*. **2025**, 245, 114250.

[S14] Q. Li, S. Li, Z. Li, W. Li, G. Qu, K. Chen, L. Huang, R. Ma, L. Deng, S. Yang, *Int. J. Biol. Macromol*. **2026**, 151161.

[S15] R. Ma, L. Xu, Z. Li, S. Li, Y. Liu, G. Qu, K. Chen, C. Chen, L. Huang, Y. Teng, *Mater. Today Bio* **2025**, 32, 101915.

[S16] H. Cheng, Z. Shi, K. Yue, X. Huang, Y. Xu, C. Gao, Z. Yao, Y. S. Zhang, J. Wang, *Acta Biomater.* **2021**, 124, 219.

[S17] M. Wang, J. Du, M. Li, F. Pierini, X. Li, J. Yu, B. Ding, *Biomater. Sci.***2023**, 11, 2383.

[S18] X. Hou, Y. Wang, M. Zhu, J. Zhang, H. Qi, M. Xu, X. Wang, *Adv. Mater. Interfaces* **2025**, 12, e00149.

[S19] W. Li, Z. Liu, X. Tan, N. Yang, Y. Liang, D. Feng, H. Li, R. Yuan, Q. Zhang, L. Liu, *Adv. Healthcare Mater.* **2024**, 13, 2304365.
